# Linking genomic evolutionary transitions to ecological phenotypic adaptations in *Spirochaetes*

**DOI:** 10.1101/2025.07.04.663154

**Published:** 2025-07-04

**Authors:** Samuel G. Huete, Killian Coullin, Elodie Chapeaublanc, Rachel Torchet, Nadia Benaroudj, Mathieu Picardeau

**Affiliations:** 1 Biology of Spirochetes Unit, Institut Pasteur, Université Paris Cité, CNRS UMR 6047, F-75015 Paris, France; 2 Bioinformatics and Biostatistics Hub, Institut Pasteur, Universite Paris Cite, F-75015, Paris, France; 3 Scientific Information Systems and Technical Expertise division, Institut Pasteur, Universite Paris Cite, F-75015, Paris, France

**Keywords:** *Spirochaetes*, evolution, pangenome, phylogenomics, adaptation, motility

## Abstract

Understanding the genetic basis of ecological adaptation is a fundamental challenge of evolutionary biology, often limited by the availability of diverse and curated datasets. *Spirochaetes* are widely distributed, ancient bacteria found in diverse environments, offering a unique opportunity to explore ecological transitions. Despite their high diversity and the presence of globally important pathogens such as syphilis (*Treponema* spp.), Lyme disease (*Borrelia* spp.), or leptospirosis (*Leptospira* spp.), *Spirochaetes* remain poorly characterized as a phylum. Moreover, the recent discovery of non-spiral lineages has broadened its complexity and require a re-evaluation of the entire phylum’s evolution. Here, we present the most comprehensive phylogenomic and functional analysis of *Spirochaetes*, examining a curated dataset of 172 spirochaetal genomes representing all cultivable spirochete species. Our robust phylogenetic framework revisits the evolutionary rooting of this phylum and reveals that the Last Spirochaetal Common Ancestor (LSCA) diverged into two major clades, with *Brachyspira* species diverging early from the rest of *Spirochaetes*. Ancestral genome reconstruction showed that the LSCA was a motile, endoflagellated bacterium with a heterotrophic metabolism, shedding light on the biology of one of the most anciently diverging bacterial phyla. Functional analysis revealed genomic signatures associated with key phenotypic adaptations within *Spirochaetes*, such as independent loss of the characteristic spiral morphology and emergence of host-associated lineages. Notably, we found that loss of endoflagellar genes correlated with the appearance of non-spiral species. Lastly, we employed phylogenetic profiling to identify previously uncharacterized motility-associated gene families, whose role was then demonstrated experimentally. Overall, this study provides new evolutionary insights into how ecological specialization has shaped spirochete genomes, offering a framework to elucidate further the mechanisms driving key evolutionary transitions in this clinically relevant phylum.

## INTRODUCTION

*Spirochaetes* (or *Spirochaetota*) form a coherent and ancient phylogenetic phylum, as initially revealed by 16S rDNA sequence comparisons (1). Until recently, all spirochetes were believed to share a unique helical or spiral-shaped morphology and utilize endoflagellar systems for motility (2). However, newly identified coccoid non-spiral *Spirochaetes* species have challenged long-standing paradigms in spirochete biology (3–7). In addition, *Spirochaetes* are widespread across diverse environments but most of the research on *Spirochaetes* thus far has focused on pathogens, including the agents of Lyme disease, syphilis and leptospirosis. The growing wealth of microbial genomic data now allows for a comprehensive exploration of spirochete diversity and evolution. This study therefore offers a unique opportunity to better understand the mechanisms of adaptation of phylogenetically related organisms to diverse lifestyles and environments. Furthermore, very few studies have systematically explored the evolution of bacterial phyla using such comprehensive approaches. Studying evolution at the phylum level offers an intermediate scale between broad tree-of-life studies and more specific genus-level analyses. This approach permits the obtention of large-scale evolutionary insights while maintaining a species-level resolution, and its potential has been previously demonstrated by uncovering the bacterial diderm-monoderm transition through the study of the *Firmicutes* phylum (8).

The phylum *Spirochaetes* comprises a single class, *Spirochaetia*, and includes four orders: *Brachyspirales*, *Brevinematales*, *Leptospirales*, and *Spirochaetales*. Among these, the order *Spirochaetales* is the largest, containing five families and numerous genera, such as *Borrelia*, *Treponema*, or *Spirochaeta* (9). *Spirochaetes* are chemo-organotrophic organisms with metabolic and respiratory requirements that vary significantly across species. They can exist as anaerobes, facultative anaerobes, microaerophiles, or aerobes. *Spirochaetes* can be free-living or hostassociated, with several pathogenic species affecting humans, other mammals, arthropods, and mollusks. For instance, symbiotic spirochetes related to treponemes are found in the hindguts of termites and wood-eating cockroaches (10). Among the human pathogens, *Borrelia* spp. include the agents of Lyme disease (*B. burgdorferi*), one of the most prevalent vector-borne infections in North America and Europe, affecting several hundred thousand people annually (11–13)*. Treponema* spp. include *T. pallidum*, the causative agent of over 50 million cases of syphilis worldwide annually (14). Leptospirosis is a re-emergent zoonotic disease caused by pathogenic *Leptospira* species and accounts for approximately 1 million severe cases and 60,000 deaths every year (15). Other diseases caused by spirochetes are relapsing fever (*Borrelia hermsii*), periodontal disease (*T. denticola*), swine dysentery (*Brachyspira hyodysenteriae*), or porcine intestinal spirochetosis (*B. pilosicoli*).

*Spirochaetes* are notably difficult to study due to their fastidious growth, recalcitrance to genetic manipulation and complex genomes. *B. burgdorferi* possesses one of the most segmented bacterial genomes, including a 1 megabase linear chromosome and over 20 plasmids (11). *T*. pallidum has a small genome of around 1.1 Mb and, until very recently, it could not be continuously cultured *in vitro* due to its strict dependence on mammalian cells, dramatically hampering progress in genetic manipulation (16,17). Another original feature is the presence of two circular chromosomes in *Leptospira* spp. (18).

Despite their clinical relevance and ecological diversity, the biology of spirochetes remains underexplored. Novel taxa, some uncultivable, are being described, but their significance remains to be elucidated (19). Since the last phylogenetic analysis of the *Spirochaetes* phylum more than 10 years ago (9), 125 new species have been described, highlighting the need for revisiting the phylum’s evolution. Here, we address this knowledge gap by providing a comprehensive study of the phylogeny and evolutionary dynamics of *Spirochaetes*. Through a robust phylogenetic study and an in-depth functional analysis, we uncovered new functional genomic signatures that shed light on the evolution of this phylum. Furthermore, using this approach, we revealed the ancestral genes present in the LSCA and discovered new motility-associated factors whose function was demonstrated experimentally.

## RESULTS

### Main features of the genomes of Spirochaetes

**Figure 1.**
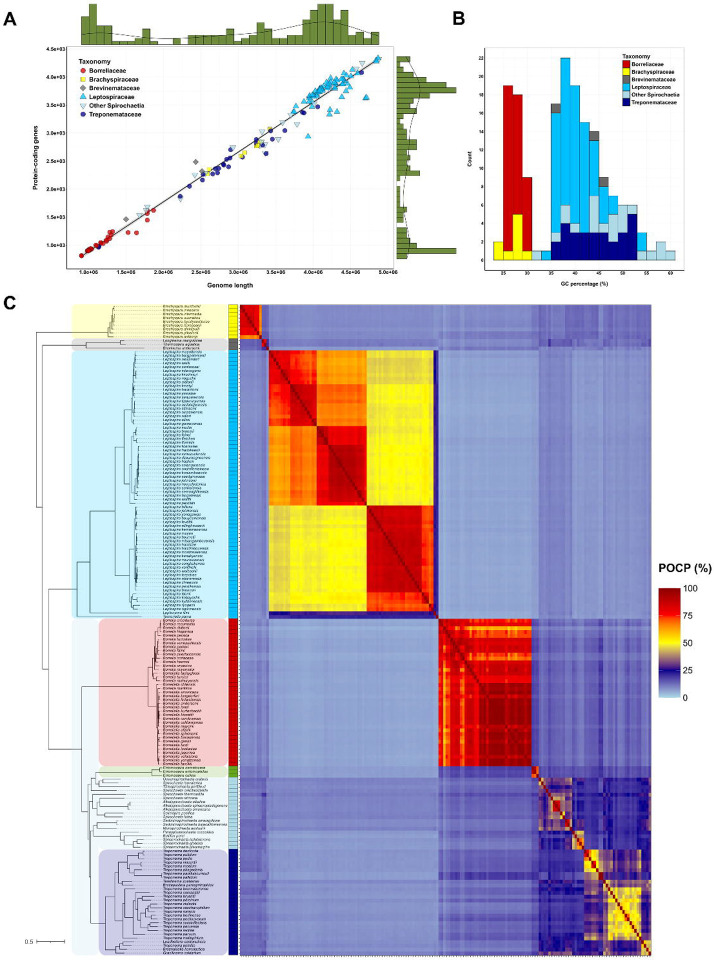
A phylogenetic framework for the *Spirochaetes* phylum (A) Correlation between genome length (in base pairs) and number of protein-coding genes in the *Spirochaetes* genomes, coloured according to the taxonomy. The green histograms on the sides represent the distribution of the genome length (upper histogram) and number of protein-coding genes (right-side histogram). (B) Distribution of GC content (%) in *Spirochaetes* coloured according to the taxonomy. (C) Phylogenetic tree of the cultivable species of *Spirochaetes*, with the main clades highlighted in colours (yellow for *Brachyspira* spp., dark grey for *Brevinematales*, cyan for *Leptospirales*, red for *Borreliaceae*, green for *Entomospira* spp., light blue for intermediate *Spirochaetia*, and dark blue for *Treponemataceae*), represented next to the pairwise matrix of Percentage of Conserved Proteins (POCP, %). The colours in the POCP matrix correspond to the continuous scale represented in the right. The coloured intermediate column represents the main clade classification. This figure is also available on the website spirochase.pasteur.cloud for dynamic exploration.

To construct a comprehensive database of cultured species within the phylum, we selected 172 genomes that ensured both representativeness and highest quality for each species. These genomes are all, except for *Borrreliella chilensis* and *Longinema margulisiae*, RefSeq curated genomes, with an average N50 of 1.37 Mb. Among them, 71 are affiliated with *Leptospiraceae*, 39 with *Borreliaceae*, 28 with *Treponemataceae*, 9 with *Brachyspiraceae*, 3 with *Brevinemataceae*, and 22 have unclear taxonomic assignments within *Spirochaetia* (Table S1). In the phylum *Spirochaetes*, genome sizes vary widely, ranging from 0.9 Mb for *Borreliella garinii* (812 proteins) to 4.9 Mb for *Spirochaeta isovalerica* (4318 proteins). Generally, the largest genomes belong to *Leptospiraceae* while the smallest genomes belong to *Borreliaceae* consistent with their tick-borne obligate parasite lifestyle (Fig 1A, Table S1). Additionally, GC percentage varies significantly, spanning from 24.1% for *Brachyspira intermedia* to 60.9% for *Spirochaeta thermophila*. Genomes from *Brachyspiraceae* and *Borreliaceae* exhibit the lowest GC content, while *Alkalispirochaeta* spp. possess the highest GC contents (Fig 1B, Table S1).

### A revisited phylogenetic framework for Spirochaetes

To study the evolution of *Spirochaetes*, we inferred orthologs using OMA and retrieved 34124 ortholog groups (OGs). Of these, only 1.47% (*N*=503) where shared by at least 50% of the species (*N*=86), highlighting the low conservation of core genes in the phylum (Fig S1). Notably, 50% of the species captured 91% of the reference pangenome (*N*=30906 OGs), illustrating the wide genetic diversity found within this phylum (Fig S1). From these OGs, 140 highly conserved soft-core genome markers were selected for phylogenetic inference. The resulting maximum-likelihood (ML) tree is well resolved under both homogeneous and heterogeneous models of evolution (Fig 1C). The topology of the unrooted phylogeny under both models was identical except for the placement of the three *Entomospira* species (Fig S2). In addition, we performed the same process using *Lindowbacteria*, the closest phylum to *Spirochaetes* (20), as a root for both the OG inference and the phylogeny. This time no discrepancies were observed between homogeneous and heterogeneous models (Fig S2). When comparing with the unrooted tree, only the pair *L. stimsonii* and *L. ainazelensis*, two closely phylogenetically related *Leptospira* species of the P1 subclade, showed different placement (Fig S3). Lastly, as an additional control of the topology, we performed a coalescence-based method that handles incomplete lineage sorting using the same soft-core markers. We found more differences between tree topologies when comparing with this method, the major being the placement of the non-spiral *Spirochaetes* species (*Bullifex porci*, *Parasphaerochaeta coccoides*, *Sphaerochaeta pleomorpha*, *Sphaerochaeta globosa* and *Sphaerochaeta halotolerans*) (Fig S4). This difference correlates with the incomplete sorting of some genes in this clade (Fig S4). However, in all phylogenies, the non-spiral *Spirochaetes* form a monophyletic clade, thus demonstrating for the first time that the spiral shape is ancestral to the phylum and was lost only once during the evolution of *Spirochaetes* (Fig S2–S4).

We thus concluded that the tree topology that best reflects the evolution of the phylum was that obtained under the rooted heterogeneous model (Fig 1C). Contrarily to previous studies that used more distant phyla to root the phylogeny (9), our results show that *Spirochaetes* separate in two large clades with the first including *Brachyspira* species and the second including the rest of *Spirochaetes* (Fig 1C). In this phylogeny, the genus *Brachyspira* is the closest to the root, forming a monophyletic clade. The three sequenced species within *Brevinematales* make a monophyletic clade where *Brevinema andersonii* is the longest branch in that clade. Similarly, the three only sequenced species within *Entomospira* make a monophyletic clade with a long separation from other *Spirochaetia* species (Fig 1C). As previously described, among the three most represented genera in *Spirochaetes* (*Leptospira* spp., *Borreliaceae* spp., and *Treponema* spp.), *Leptospira* and *Borreliaceae* form monophyletic clades subdivided in four and two major subclades, respectively (Fig 1C) (21,22). However, the classification of the clade containing *Treponema* species is less clear. While all *Treponemataceae* species form a monophyletic clade alongside the recently described *Breznakiellaceae* family (23,24), the naming of some of these genera may lead to confusion. Based on this and previous evidence we propose reclassifying the species *Treponema primitia* to a different genus and renaming *Teretinema zuelzerae* and *Brucepastera parasyntrophica* to *Treponema zuelzerae* and *Treponema parasyntrophica*, respectively. Such change would imply that termite gut treponemes in the *Breznakiellaceae* family would conform a basal clade to all *Treponema* spp. which would then separate in two main subclades (Fig 1C). To further clarify the status of *T. zuelzerae* and *B. parasyntrophica*, we performed another phylogenetic inference restricted to the *Treponematales* clade. This increased by over 3-fold the number of phylogenetic markers (*N*=440), thus representing the most robust phylogenetic inference performed to date in *Treponema* species. Our data confirmed that *Treponema* species subdivide in two main subclades, the first (T1) containing *T. pallidum* and *T. denticola* alongside *T. zuelzerae* and *B. parasyntrophica*, among other treponemes, and the second (T2) containing *T. maltophilum* and *T. porcinum*, among other treponemes (Fig S5) (23,24).

### Pairwise comparisons support the phylogenetic analysis

Pairwise comparison analyses using average nucleotide identity (ANIb, Fig S6), kmer-based tetranucleotide method (TETRA, Fig S7), and the percentage of conserved proteins (POCP, Fig 1C), all indicated a good correlation with the phylogeny presented in Fig 1C. As previously described for other prokaryotes (25), POCP was the best predictor of genus boundaries within *Spirochaetes* (Fig S8). All *Brachyspira* spp. and *Borreliaceae* spp. share a POCP ≥ 50%, thus clearly assigning them within a single genus. However, not all *Leptospira* spp. nor *Treponema* spp. share POCP values above 50%, thus reflecting that these genera include a higher diversity of species than *Brachyspira* and *Borreliaceae* (Fig 1C, Fig S8, Table S2). When looking at POCP values, two major clusters are found within *Treponema* spp., the first including a subset of the T1 subclade (*T. denticola*, *T. putidum*, *T. pedis*, *T. vincentii*, and *T. medium*, Fig 1C), and the second including all T2-subclade treponemes (Fig 1C, Fig S8). In addition, our results suggest that *Longinema margulisiae* and *Thermospira aquatica*, two *Brevinematales*, should be renamed to be included in the same genus. This applies as well to *T. primitia* and *Leadbettera azotonutricia* (Fig 1C, Table S2). In general, we propose a 45% POCP threshold for genus assignment within the *Spirochaetes* phylum (Fig S8). However, genus nomenclature within *Spirochaetes* should be treated with caution since it represents different levels of diversity for the same taxonomic status.

Altogether, these results present the most updated phylogeny of the phylum *Spirochaetota* to date, supporting an evolutionary scenario where the Last Spirochaetal Common Ancestor (LSCA) gave rise to two main clades, separating *Brachyspira* species from the rest of *Spirochaetes* early in the phylum’s evolution.

### Cluster-based annotation improves functional characterization in Spirochaetes

To understand the functions encoded by each species, we annotated the OGs using a clusterbased approach with fine-grained orthology (see Methods). Each sequence of the 491 most prevalent OGs (63423 proteins, 14.5% of the total dataset) was annotated, showing that 96.3% of the annotated sequences had consistent functions (COG categories) within their OG, thus validating our approach. This allowed to assign a COG category to 58% (*N*=19893) of all OGs, with an average 70% annotation level per species (ranging 48–87%) (Fig S9). In addition, we successfully assigned a maximum annotation level to 83% (*N*=28389) of all OGs, with an average 88% annotation level per species (ranging 64–100%) (Fig S9). Interestingly, *Leptospira* spp. had a significantly higher number of OGs of eukaryotic origin than the other *Spirochaetes*, some of which are related to the regulation of key cellular processes like actin cytoskeleton dynamics, growth regulation or transcriptional regulation (Table S3). In addition, *Borreliaceae* spp. had a significantly higher number of OGs of viral origin than other *Spirochaetes*, consistent with the abundance of phage-derived plasmids in these species (26) (Fig S10).

### Functional classification of genomes differentiates key phenotypes in the phylum

**Figure 2.**
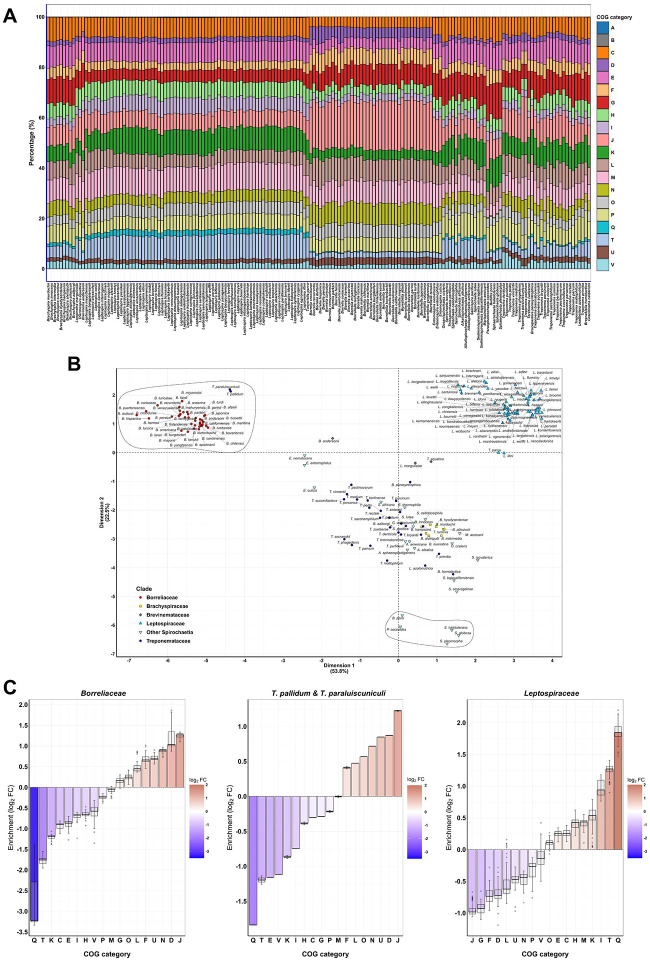
Functional genomic signatures of phenotypic adaptation. (A) Genome repartition by COG categories, excluding unknown function genes. The genomes are ordered from left to right according to their phylogenetic position in Fig 1C, and COG categories are color-coded and ordered alphabetically from top to bottom as represented in the legend. This figure is also available on the website spirochase.pasteur.cloud for interactive exploration. (B) Principal Component Analysis (PCA) individuals plot of the COG category distribution, with the main taxonomic clades coloured as indicated in the legend. The clusters containing the host-dependent species (*Borrelia* spp., *T. pallidum* and *T. paraluiscuniculi*) (upper-left position) and the non-spiral *Spirochaetes* (lower position) are circled with a dashed line. (C) Enrichment analysis of the COG categories against all other *Spirochaetes* species of the *Borreliaceae* family, *T. pallidum* and *T. paraluiscuniculi* (excluding *Borreliaceae* spp.), and the *Leptospiraceae* family. Enrichment values (expressed as log_2_FC) are arranged by increasing order (left-to-right) and coloured according to the colour gradient on the left of each plot. The boxplots indicate the log_2_FC median and the first and third quartiles for all evaluated species. COG category letters correspond to: [A] RNA processing and modification, [B] Chromatin structure and dynamics, [C] Energy production and conversion, [D] Cell cycle control, cell division, chromosome partitioning, [E] Amino acid transport and metabolism, [F] Nucleotide transport and metabolism, [G] Carbohydrate transport and metabolism, [H] Coenzyme transport and metabolism, [I] Lipid transport and metabolism, [J] Translation, ribosomal structure and biogenesis, [K] Transcription, [L] Replication, recombination and repair, [M] Cell wall, membrane, envelope biogenesis, [N] Cell motility, [O] Posttranslational modification, protein turnover, chaperones, [P] Inorganic ion transport and metabolism, [Q] Secondary metabolites biosynthesis, transport and catabolism, [T] Signal transduction mechanisms, [U] Intracellular trafficking, secretion, and vesicular transport, and [V] Defense mechanisms.

We then analysed the percentage of the genome devoted to a given function (COG category) and observed distinct functional signatures among different clades (Fig 2A). Principal component analysis (PCA) evidenced that 81% of the functional variability of this phylum is explained by two components (PC1 and PC2), with only RNA processing and modification (A) and chromatin structure and dynamics (B) having a low contribution (1.90–1.96%) to these components (Fig S11).

This is because A and B categories have a low representation in *Spirochaetes*, with *Leptospira* being the genus with most genes devoted to chromatin regulation (B, 0.19–0.28%), consistent with their unique capacity to organize the genome around chromatin-like structures (27). *Brachyspira* is the genus with most genes devoted to RNA modification (A, 0.05–0.21%) (Fig 2A, Table S4). In contrast, transcription (K), signal transduction (T) and energy production (C) were the categories that contributed the most to these components (5.95%, 5.83% and 5.73%, respectively, Fig S11). Importantly, the category that contributed the most to the second component (PC2) was secondary metabolism (Q, 16.28%). This evidences that the percentage of the genome devoted to transcription, signal transduction, energy production and secondary metabolism are the most defining functional features within *Spirochaetes* (Fig 2A, Fig S11).

PCA analysis of the individuals revealed clusters of species that did not fully correlate with the phylogenetic signal. Instead, certain key phenotypes such as non-spiral spirochetes or hostdependent species formed distinct clusters, well separated from their close relatives. *Treponema pallidum* and *Treponema paraluiscuniculi* clustered together with *Borreliaceae* genomes, all of them being host-dependent species that have adopted a parasitic lifestyle (Fig 2B). While the three of them show lower genome sizes than other *Treponemataceae* or *Brevinemataceae* species, this clustering cannot be solely attributed to the small genomes, since *Entomospira* spp. or *Brevinematales* have smaller genome sizes than some *Borreliaceae* and they cluster independently (Fig 1A, Fig 2B). Furthermore, all five species known to lack the characteristic spiral shape of *Spirochaetes* clustered together and separately from other *Spirochaetia* species of their same clade (Fig 2B). All *Leptospira* spp. clustered independently as well (Fig 2B).

### Host-dependent species are enriched in translation and depleted in transcription categories

Once we identified functional signatures in the *Spirochaetes* phylum, we proceeded to characterizing them using enrichment analysis on the COG categories. Host-dependent species had a significant enrichment of categories devoted to translation (J) and cell cycle control (D), as well as a decrease in secondary metabolism (Q), signal transduction (T) and transcription (K) categories (Fig 2C). On the contrary, *Leptospiraceae* had a significant enrichment in secondary metabolism (Q) and chromatin dynamics (B), and a decreased percentage of genes devoted to translation (J) or carbohydrate metabolism (G) (Fig 2C). This correlates with previous studies showing that the *Leptospira* genus are the only spirochetes encoding for histones that participate in chromatin organization (27). Non-spiral spirochetes had a dramatic decrease in the motility category (N, log_2_FC of −3.8), along with an increase in carbohydrate transport and metabolism (G) (Fig S12). Interestingly, almost no gene belonging to the N category was present in any of the non-spiral spirochete genomes, thus evidencing that the loss of the spiral shape correlated with a motility loss as well (Fig 2A, Table S4).

To further illustrate the strength of our classification strategy, we employed the same functional classification for just the *Leptospira* genus. To date, no study could *a priori* distinguish highly virulent leptospires (P1+ group) from non-pathogenic leptospires of the P clade (P1- group and P2 subclade) based solely on genomic features. COG categories distribution revealed that P1+ *Leptospira* spp. possess a different signature than the rest of the *Leptospira* genus based on their functional genomic content (Fig S12). All P1+ species clustered together, while P1- species clustered with P2 species despite belonging to a different phylogenetic subclade (Fig S12). Interestingly, *L. borgpetersenii* serovar Hardjo-Bovis, the most host-dependent *Leptospira* species (28), showed the most differential separation from all pathogenic leptospires (Fig S12).

Overall, we demonstrate that functional genomics, here defined as the functional repartition of the genome in different biological processes, is a key signature distinguishing the different lifestyles of *Spirochaetes*.

### Ancestral genome reconstruction reveals that the Last Spirochaetal Common Ancestor was an heterotrophic spiral-shaped bacteria

**Figure 3.**
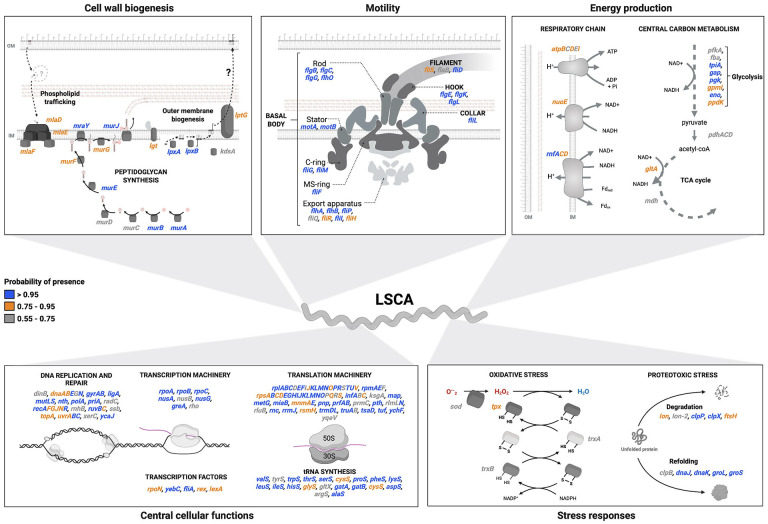
Features of the Last Spirochaetal Common Ancestor (LSCA). Schematic representation of the metabolism and selected features of the LSCA as inferred from the OGs present in the root. Each gene name is coloured according to their probability of presence indicated in the legend on the left-side. Each of the five panels represents a subset of genes with related functions. Question marks indicate missing functions classically needed for the pathways of cell wall biogenesis.

To further investigate the evolution of *Spirochaetes*, we used a ML-based approach to identify OGs present in the ancestral root of the phylogeny (Fig 1C). Among the 34124 OGs analyzed, 511 OGs (1.5%) were inferred as ancestral to the whole phylum, with a probability above 80% for 60% of them (*N*=304) (Fig S13, Table S5). The most represented functions (31%, *N*=159) were housekeeping processes such as DNA replication (L), transcription (K) and translation (J) (Fig 3). We also identified 23 ancestral flagellar proteins, with a probability >95% for 78% of them. Even though genes encoding the L- and P-ring flagellar sections were absent, this indicates motility in the LSCA (Fig 3, Table S5).

Additionally, genes involved in cell wall biogenesis, including peptidoglycan production (*murABCDEFGJ*, *mraY*), enzymes of lipid A biosynthesis (*lpxAB*, *lgt*, *kdsA*, *htrB*), components of the Maintenance of Lipid Assymetry pathway (*mlaDEF*), and a homolog of the LPS permease LptG, were identified, most with probabilities above 80–90% (Fig 3). Cytoskeletal proteins associated with cell curvature (*mreB, ccmA*) were also found to be present (probability >98%), indicating that the LSCA was spiral-shaped (29,30). The presence of genes involved in metabolic pathways, such as glycolysis, TCA cycle, and the respiratory chain, indicate that the LSCA was a heterotrophic bacterium capable of central carbon metabolism (Fig 3). Lastly, genes coding for factors of the oxidative (*sod*, *tpx*, *trxAB*) and proteotoxic (*dnaKJ*, *groEL*, *clpB*) stress responses were also found alongside two heme-related enzymes (*hemNH*). Overall, our analysis reveals that the LSCA was a motile, diderm, spiral-shaped, and heterotrophic bacterium capable of sustaining most housekeeping functions found in nowadays spirochetes.

### Phylogenetic profiling helps uncover new motility factors

Through the present study, we have generated an annotated ortholog database that is made available to the community interested in spirochete research in the site spirochase.pasteur.cloud. To illustrate the combined versatility of these datasets, phylogenetic profiling was used to generate functional predictions over previously uncharacterized genes. We searched for genes that were conserved in most spirochete species but absent in the non-spiral spirochete species. Although most of them (≈ 60%) were related to motility (Fig S14, Table S6), two highly conserved and uncharacterized gene families were identified: OG8135 (WP_002706277.1) and OG9070 (WP_013945094.1). We thus hypothesized that these genes may be linked to motility in *Spirochaetes*.

**Figure 4.**
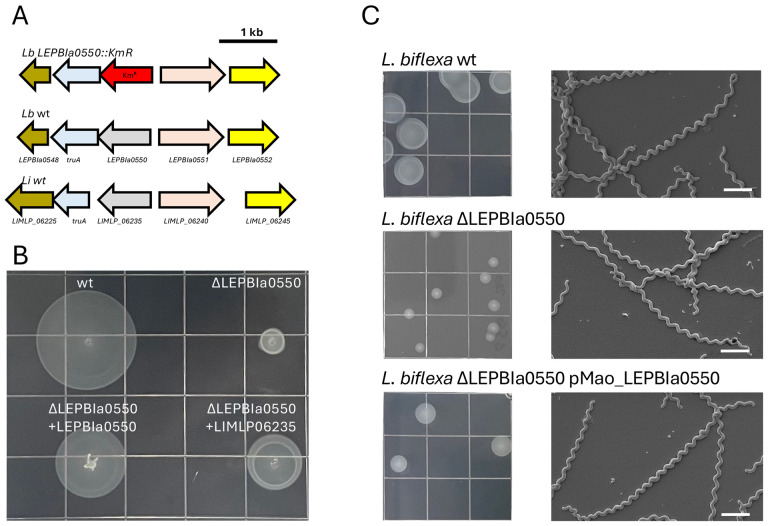
A novel family of previously uncharacterized motility-related factors in *Spirochaetes*. (A) Schematic representation of LEPBIa0550 locus in model spiral-shaped *Leptospira spp*.; *L. biflexa* serovar Patoc strain Patoc 1 (*Lb wt*), *L. biflexa* LEPBIa0550::KmR (*Lb* Δ*LEPBIa0550), and L. interrogans* serovar Manilae strain L495 (*Li wt*). (B) Motility phenotype of *L. biflexa* serovar Patoc strain Patoc 1 (wt), mutant (Δ*LEPBIa0550)* and complemented strains (Δ*LEPBIa0550*+*LEPBIa0550* and Δ*LEPBIa0550*+*LIMLP02635*) onto soft agar plates (0.8%). (C) Colony (left panel) and cell morphologies of *L. biflexa wt*, *L. biflexa* mutant Δ*LEPBIa0550* and complemented strain *L. biflexa* Δ*LEPBIa0550* + *LEPBIa0550*. Cell morphology was determined by scanning electron micrograph of cells. The bar in the figures indicates 1 μm.

To determine whether these previously unannotated OGs were linked to motility, we attempted to inactivate their orthologs in the model species *Leptospira biflexa* (S1 subclade of the *Leptospira* genus, Fig 4A). Despite several attempts, we were unable to delete the LEPBIa2229 gene (OG9070) by allelic exchange, thus suggesting that this gene is essential. However, we successfully inactivated LEPBIa0550 (OG8135) and the resulting mutant exhibited a motility defect compared to the WT *L. biflexa* (Fig 4BC). Gene inactivation of LEPBIa0550 does not affect bacterial growth kinetics in liquid medium, thus the observed differential colony diameter between these strains can only be attributed to differences in motility within semisolid media (Fig 4B). Partial complementation was achieved by the expression of the LEPBIa0550 gene or its homologue from *L. interrogans* (LIMLP02635) (Fig. 4). Interestingly, inactivation of LEPBIa0550 did not lead to morphological defects as assessed by scanning electron microscopy (Fig 4C). This demonstrates that this highly conserved gene family, conserved in all spiral-shaped *Spirochaetes* and belonging to the larger uncharacterized domain DUF2225, plays a role in the motility of *Spirochaetes*.

## DISCUSSION

Despite syphilis, Lyme disease and leptospirosis being emerging or re-emerging diseases, little is known about the biology of spirochetes compared to model bacteria. Spirochete research in both pathogenic and non-pathogenic species has traditionally been hampered by fastidious growth, limited genetic tools and incomplete genome annotations, complicating the progress of hypothesis-driven studies following omics analyses (31,32).

Here, we significantly improved the functional annotation of spirochaetal proteins by applying a cluster-based annotation approach, achieving an average 70% annotation level per species and nearly 90% annotation for some genomes. This represents a significant improvement (15%) over previous studies, where, for example, only 44% of the *L. interrogans* genome was annotated, compared to 64% in our analysis (18,33). In addition, we assigned putative origin (bacterial, eukaryotic, viral or archaeal) and closest ortholog for 60% (*N*=8496) of unknown function genes (S), reducing the proportion of orphan genes in *Spirochaetes* to just 16% (*N*=5735), a significant advancement for the field.

We also constructed a robust phylogeny for *Spirochaetes*, supported by multiple comparative genomic methods. This revisited phylogeny challenges previous views of *Spirochaetes* by redefining the evolutionary rooting of the phylum, likely due to increased species diversity and better selection of the outgroup (Fig 1C) (9). Such phylogenetic framework also provides a basis for revising the taxonomy of the phylum. We propose a 45% POCP threshold for defining genera within *Spirochaetes*, which could resolve a long-standing controversy over genus classification (34–36). We strongly recommend either standardizing genus nomenclature across the phylum or, alternatively, recognizing that “genus” within *Spirochaetes* represents varying levels of species divergence. For example, in the current formulation, the *Treponema* genus is significantly more diverse than *Leptospira*, and *Leptospira* itself encompasses greater diversity than the *Borreliaceae* family (which includes *Borrelia* and *Borreliella*).

Our comparative genomic approach offers a valuable resource for characterizing unknown gene families. As proof of concept, this strategy helped us link two previously orphan gene families (OG8135 and OG9070) to motility. A role of the OG8135 family in motility could be demonstrated experimentally. Thus, the datasets developed in this study represent a useful framework for building hypothesis-driven studies allowing functional characterization of previously orphan gene families. To facilitate phylogenetic profiling and access to the datasets, we created an interactive online resource (spirochase.pasteur.cloud) allowing researchers to explore gene distributions within *Spirochaetes*.

Our analyses also explored how functional repartition of the genome correlated with species evolution. We revealed that genome organization expressed as the relative percentage of the genome devoted to a particular function correlated with key phenotypic adaptations. While this may be a universal signature in microbes, we observed that host-dependent species dedicate a larger proportion of their genome to translation and less to transcription. This indicates that parasitic microorganisms have shifted genome regulation towards reducing transcription factors but increasing post-transcriptional regulation, as evoked by *Borrelia* spp. (37–39). Additionally, we demonstrated that genome function distribution is as well the most differential genomic feature between highly-virulent *Leptospira* species (P1+ group) versus other leptospires, facilitating further research in this direction. Until now, no other *in silico* classification method could demonstrate that highly virulent P1+ species are different from other *Leptospira* without *a priori* phenotypic information (27).

Our study uncovers the metabolism and characteristics of the Last Spirochaetal Common Ancestor, providing insights into the biology of the earliest spirochetes. We reveal that the LSCA was a diderm, spiral-shaped, and heterotrophic bacterium with a nearly complete flagellar system, lacking only the L- and P-ring components. Their absence correlates with the observation that only *Leptospira* spp. of all *Spirochaetes* possess these structures (2), and suggests that the LSCA flagellum was periplasmic (40,41). Furthermore, the presence of a partial LPS biosynthesis and export pathway does not allow to conclude for the presence of LPS in the LSCA. The only known LPS-containing *Spirochaetes* are *Leptospira* spp (42). Notably, the LSCA possessed a SOD-Trx system for oxidative stress resistance, consistent with the studies estimating *Spirochaetes* emergence around the Great Oxidation Event (2300 MYA) (43). Additionally, the presence of two heme-related enzymes (*hemNH*) suggests that the LSCA had a partial heme metabolism, which was lost in modern *Borrelia* and *Treponema* spp. (44).

As more metagenomes from uncultivated species are made available, additional studies will be required to revisit the framework presented here and further reshape our current views of spirochete evolution. Overall, our study represents a major step forward in our understanding of *Spirochaetes*, offering new insights and facilitating further research to decipher the evolution of this medically and environmentally important bacterial phylum.

## METHODS

### Data collection

176 genomes covering all cultivable species within the *Spirochaetes* phylum were downloaded from the National Center for Biotechnology Information (NCBI) as of August 2024 following manual curation. The genomes corresponding to the species *Alkalispirochaeta odontotermitis* (GCF_000768055.1), *Rectinema subterraneum* (GCF_009768935.1), *Treponema lecithinolyticum* (GCF_000468055.1), and *Marispirochaeta associata* (GCA_001749745.1) were removed because of high level contamination with other organisms. For the selection of genomes, one genome was chosen per species TaxID in NCBI prioritizing the RefSeq-validated genome per TaxID. For the two species in which there was no RefSeq-validated reference genome (*Borrreliella chilensis* and *Longinema margulisiae*), we inspected the literature and selected the only available GenBank genome after checking completeness and contamination. For the reference genomes of the 69 *Leptospira* spp., we followed the latest Position Statement of the International Leptospirosis Society (ILS) (45) and the International Committee on Systematics of Prokaryotes Subcommittee on the taxonomy of *Leptospiraceae* (46). It should be noted that there is no publicly available genome for the genus *Cristispira*. The final selected number of genomes is 172 and their description and assembly numbers are available in Table S1.

### Orthology inference

For the orthology inference, we employed the protocol described in (47) with a few modifications. We used OMA standalone version 2.5.0 (48) with the exported reference proteomes. To ensure that orthologs were computed without bias, in a first run we used only the 172 pre-selected proteomes with the following parameters: estimated species tree, bottom-up inference of HOGs, mid-point rooting, minimum score of 181, length tolerance ratio of 0.61, stable pair tolerance of 1.81, inparalog tolerance of 3.0, verified pair tolerance of 1.53, minimum sequence length of 50, and minimum edge completeness of 0.65. In a second run, we used the 172 pre-selected proteomes along with two *Lindowbacteria* species (GCA_001782795, GCA_001784175) used as outgroup for rooting, with the same parameters as above except for the rooting. For the orthology inference of *Treponematales*, we used the 28 pre-selected genomes belonging to the *Treponemataceae* clade (Table S1) alongside three of their closest relatives for the rooting (GCF_000143985.1, GCF_000378205.1, GCF_002087085.1) (Fig 1C) with the same parameters as above.

Calculation of the pangenome accumulation curve was obtained following the method described in (49), using 100 random iterations in the presence/absence matrix of OGs. The regression curve was calculated using a generalized additive model (gam) with a cubic spline under the formula y ~ s(x, bs = “cs”).

### Tree inference

Tree inference was determined as described in (47) with a few modifications. Briefly, we first selected 140 highly-conserved soft-core genome markers (95% conservation, 163 species) from the first run of OMA OGs using the filter_groups.py script from (47) and aligned them individually with MAFFT version 7.505 under the L-INS-i algorithm (50). We then soft-trimmed the alignments individually using BMGE version 1.12 (51) under the BLOSUM30 matrix with the following parameters: maximum entropy threshold of 0.95, sliding window size of 1, minimum length of selected regions of 1. Lastly, we concatenated the trimmed alignments to build the supermatrix using the concat_alignments.py script available in (47). This supermatrix was then used as input for IQ-TREE version 2.3.2 (52) with 10000 ultra-fast bootstraps and 10000 SH-alrt (Shimodaira-Hasegawa-like procedure test) (53) under the homologous best-fit bacterial model of evolution LG+F+I+R10. The tree generated was then used as a guide tree for a second tree inference under the heterogeneous model of evolution LG+C20+R10 with IQ-TREE and the same parameters. The same process was repeated with the second run of OMA OGs including the two *Lindowbacteria* genomes (see above) with 130 highly-conserved soft-core genome markers (95% conservation, 165 species).

For the coalescence-based method, we used the same 130 trimmed soft-core genome marker alignments including the two *Lindowbacteria* genomes (see above) and inferred individual phylogenies using FastTree version 2.1.11 (54). We then used these individual phylogenies to infer the final species tree using ASTRAL version 5.7.8 (55).

For the Treponematales phylogenetic inference, we used 440 soft-core genome markers (95% conservation, 29 species) obtained from the orthology inference (see above) and proceeded as before to obtained the supermatrix. This supermatrix was then used as input for IQ-TREE version 2.3.2 with 10000 ultra-fast bootstraps and 10000 SH-alrt (Shimodaira-Hasegawa-like procedure test) under the homologous best-fit bacterial model of evolution LG+F+I+R10.

To support the phylogenetic tree, we calculated pairwise comparisons of Percentage of Conserved Proteins (POCP), Average Nucleotide Identity (ANI) and a kmer-based method (TETRA). ANI (with the BLASTn module) and TETRA were calculated with PyAni version 0.2.11 (56). POCP was calculated with POCP-nf version 2.2.0 (25,57).

### Functional annotation

Functional annotation of the proteomes was determined using a cluster-based approach with fine-grained orthology. Briefly, we aimed at obtaining the centroid of each OG by clustering each OG group from the OMA orthology inference (see above) using MMseqs2 version 15–6f452 (58) and obtaining a single representative sequence per OG with the following parameters: workflow easy-cluster, infinite e-value, minimum sequence identity 0, coverage mode 1, alignment coverage 0. Each representative sequence was then searched with eggNOG-mapper version 2.1.12 (59) on eggNOG DB version 5.0.2 (60). Sequence searches were performed using four consecutive approaches: DIAMOND ultra-sensitive (61), HMMER with database 2 (bacteria), HMMER with database 2157 (archaea), and HMMER with database 10239 (viruses) (62). We then assigned an annotation to each OG according to the following hierarchy: if DIAMOND search provided a COG category different than “unknown” (S/NA) it was retained, otherwise we looked for the unannotated OGs in the HMMER searches (first bacterial, then archaeal, lastly viruses). The DIAMOND search provided an annotation for 53.5% of all OGs, and the HMMER searches complemented this to 58.2% of all OGs. For assignment of the maximum annotation level following eggNOG-mapper, the same hierarchical procedure was followed.

Principal Component Analysis (PCA) was performed with the PCA function of the FactoMineR package (63) in R version 4.3.0 (64) with the normalized percentage of known COG categories per genome as input. Log_2_FC values were calculated as the logarithmic base 2 ratio between the percentage of OGs within a species’ genome annotated as a given COG category and the percentage of OGs within all other species’ genomes annotated as the same COG category unless otherwise stated.

### Ancestral genome reconstruction

The genomic components present in the LSCA were reconstructed using PastML version 1.9.41 (65) with a combination of the presence/absence matrix of OGs and the phylogeny presented in Fig 1C. To ensure a robust inference, ancestry prediction was performed in two steps: first, we run PastML with a marginal posterior probabilities approximation (MPPA method) to determine the maximum-likelihood model parameters and marginal probabilities for each column under the F81 model (66); then, we run PastML again using these pre-determined model parameters to evaluate the most likely evolutionary scenario and produce an internally consistent result for all nodes under the JOINT method (67). Therefore, the final root status is the one obtained under the JOINT prediction and the probabilities presented are the ones obtained under the MPPA prediction. For the final analysis, only OGs with JOINT-based root status “presence” and an MPPA-based probability ≥55% were retained.

### Phylogenetic profiling

To obtain the OGs lost by the non-spiral *Spirochaetes* (*Bullifex porci*, *Parasphaerochaeta coccoides*, *Sphaerochaeta pleomorpha*, *Sphaerochaeta globosa* and *Sphaerochaeta halotolerans*), we first looked for OGs that were completely absent from those species but present in 80% or more of the other *Spirochaetes* phylum species (Type = “ABSENT”). As a complementary strategy, we also looked for OGs completely absent from the same species but highly conserved (≥95% conservation) in the closest clade species (all *Treponemataceae* spp., *Alkalispirochaeta alkalica*, *Alkalispirochaeta americana*, *Alkalispirochaeta sphaeroplastigenens*, *Marispirochaeta aestuarii*, *Salinispira pacifica*, *Sediminispirochaeta bajacaliforniensis*, *Sediminispirochaeta smaragdinae*, *Spirochaeta africana*, *Spirochaeta lutea*, and *Spirochaeta thermophila*) (Type = “LOST”). Both datasets were then combined and are available in Table S6.

### Gene inactivation in *L. biflexa* and phenotypic characterization of mutant strains

*Leptospira biflexa* strains were grown in liquid, solid (1% agar plates) or semi-solid (0.8% agar plates) Ellinghausen-McCullough-Johnson-Harris (EMJH) at 30°C. For targeted mutagenesis of LEPBIa0550 and LEPBIa2229 in *L. biflexa*, a kanamycin resistance cassette replacing the coding sequence of LEPBIa0550 and 0.9 kb sequences homologous to the sequences flanking the target gene was synthesized by GeneArt (Life Technologies) and cloned in an *E. coli* vector. Plasmid DNA was then introduced in *L. biflexa* serovar Patoc by electroporation as previously described (68) with a Biorad Gene Pulser Xcell. Electroporated cells were plated on EMJH agar plates supplemented with 50 μg/ml kanamycin. Plates were incubated for 1 week at 30 °C and kanamycin-resistant colonies were screened for double crossing-over events by PCR using primer pairs LEPBa550_1 (5’-TGCCATTCATTCCGTTTCCG-3’) and LEPBa550_2 (5’-CTGTATCGGTCCTTCCAGCC-3’), and LEPBa_2229_1 (5’-TCAGGGTCAGCCTACGTCGG-3’) and LEPBa_2229_2 (5’-CCTGAGTCCCGAACGGGACG-3’). To construct the plasmids for complementation of LEPBIa0550 mutant, LEPBIa0550 (5’-AAGAGCTCTTCCCCACGGAAGTTTTTAACG-3’ and 5’-AATCTAGAGCCAAGTGCCGATTCGATCGAAG-3’), and *L. interrogans* (LIMLP_02635) (5’-AAGAGCTCTGTTGATTCTGTCCACCAGG-3’ and 5’-AATCTAGAAATGTGGATTTCTTCCTTGAGAAG-3’) were amplified, digested (restriction sites for SacI, XbaI or NotI are underlined), and cloned into pMaORI (69). Whole plasmid sequencing was performed by Plasmidsaurus using Oxford Nanopore Technology with custom analysis and annotation. Conjugation was performed as previously described (70). Briefly, *E. coli* β2163 containing plasmid of interest was incubated with log-phase *L. biflexa* on a membrane filter and placed on EMJH plate supplemented with 0.3 mM diaminopimelic acid and incubated for 16–20 h at 30°C. The bacteria were then resuspended in EMJH and spread onto EMJH solid agar plates supplemented with 50 μg/ml spectinomycin. The plates were incubated at 30°C until leptospiral colonies were observed at 1 week for *L. biflexa*. Motility is evaluated by plating mid-log phase *Leptospira* strains into 0.8% soft agar plates. Colony expansion diameters were measured at 7 days post-inoculation. For Scanning Electron Microscopy, bacteria were fixed in 2.5% glutaraldehyde 2 hours at room temperature and overnight at 4°C, postfixed for 1.5 hours in 1% osmium. Samples were then dehydrated through a graded ethanol series followed by critical point drying with CO2 (CPD300, LEICA). Dried specimens were gold/palladium sputtercoated (20 nm) with a gun ionic evaporator ACE 600, LEICA. The samples were imaged in a JEOL IT700HR field emission scanning electron microscope.

## Supplementary Material

Supplement 1 **Figure S1. Pangenome analysis of the phylum *Spirochaetes*.** (A) Pangenome accumulation plot of the *Spirochaetes* phylum representing the cumulative number of different OGs. This was calculated using 100 random iterations in the presence/absence matrix of OGs. Each blue dot represents one iteration, and the black line is the smooth curve of regression calculated using a generalized additive model (gam) with a cubic spline under the formula y ~ s(x, bs = “cs”). (B) Cumulative (green dots) and non-cumulative (blue dots) numbers of orthologs shared as the number of species increases in the range 2 to 172. The Y axis is represented in logarithmic scale to facilitate visualization.
**Figure S2. Phylogenetic comparisons of the *Spirochaetes* phylum (I).** (A) Co-phylo plot representing the comparison between the phylogeny obtained under the unrooted homogeneous model of evolution (LG+F+I+R10, left side) and the unrooted heterogeneous model of evolution (LG+C20+R10, right side). Red lines connect the same leaves (species) in both trees. (B) Co-phylo plot representing the comparison between the phylogeny obtained under the rooted homogeneous model of evolution (LG+F+I+R10, left side) and the rooted heterogeneous model of evolution (LG+C20+R10, right side). Red lines connect the same leaves (species) in both trees.
**Figure S3. Phylogenetic comparisons of the *Spirochaetes* phylum (II).** (A) Co-phylo plot representing the comparison between the phylogeny obtained under the rooted homogeneous model of evolution (LG+F+I+R10, left side) and the unrooted homogeneous model of evolution (LG+F+I+R10, right side). Red lines connect the same leaves (species) in both trees. (B) Co-phylo plot representing the comparison between the phylogeny obtained under the rooted heterogeneous model of evolution (LG+C20+R10, left side) and the unrooted heterogeneous model of evolution (LG+C20+R10, right side). Red lines connect the same leaves (species) in both trees.
**Figure S4. Phylogenetic comparisons of the *Spirochaetes* phylum (III).** (A) Co-phylo plot representing the comparison between the phylogeny obtained under the rooted heterogeneous model of evolution (LG+C20+R10, left side) and the rooted multi-species coalescence model of evolution (ASTRAL, right side). Red lines connect the same leaves (species) in both trees. (B) Presence/absence plot of the 140 soft-core genome markers (OGs) used for the phylogenetic inference, with species in which the OG is present marked in blue and absent in grey. The Y axis contains the species ordered from top to bottom according to their phylogenetic position in Fig 1C.
**Figure S5. An updated phylogeny for the *Treponematales* order.** Phylogeny of the *Treponematales* order obtained under the model LG+F+I+R10 (best-fit bacterial model for *Spirochaetes*) using 440 soft-core genome markers and rooted using the closest clade in Fig 1C (*Sediminispirochaeta smaragdinae*, *Sediminispirochaeta bajacaliforniensis*, *Marispirochaeta aestuarii*). The two subclades within the *Treponema* genus are highlighted in blue (T1 subclade) and grey (T2 subclade). All nodes had SH-alrt and bootstrap support values of 100% except for the one splitting *T. parvum* from *T. socranskii*, *T. porcinum* and *T. bryantii*, which had 97/91% support.
**Figure S6. Average nucleotide identity matrix of the *Spirochaetes*.** Phylogenetic tree of the cultivable species of *Spirochaetes*, with the main clades highlighted in colours (yellow for *Brachyspira* spp., dark grey for *Brevinematales*, cyan for *Leptospirales*, red for *Borreliaceae*, green for *Entomospira* spp., light blue for intermediate *Spirochaetia*, and dark blue for *Treponemataceae*), represented next to the pairwise matrix of Average Nucleotide Identity using the BLASTn method (ANIb, %). The colours in the ANIb matrix correspond to the continuous scale represented in the right. The intermediate coloured column represents the main clade classification.
**Figure S7. Kmer-based tetranucleotide matrix of the *Spirochaetes*.** Phylogenetic tree of the cultivable species of *Spirochaetes*, with the main clades highlighted in colours (yellow for *Brachyspira* spp., dark grey for *Brevinematales*, cyan for *Leptospirales*, red for *Borreliaceae*, green for *Entomospira* spp., light blue for intermediate *Spirochaetia*, and dark blue for *Treponemataceae*), represented next to the pairwise matrix of a kmer-based tetranucleotide method (TETRA, %). The colours in the TETRA matrix correspond to the continuous scale represented in the right. The intermediate coloured column represents the main clade classification.
**Figure S8. Percentage of conserved proteins as predictor of genus boundaries in *Spirochaetes*.** (A) Histogram of distribution of Average Nucleotide Identity using the BLASTn method (ANIb, %) values for all pairwise comparisons of the phylum *Spirochaetes*. The distribution of the values is a single mode skewed uniformly to the right. (B) Histogram of distribution of Percentage of Conserved Proteins (POCP, %) values for all pairwise comparisons of the phylum *Spirochaetes*. The values are distributed in three main modes with clear boundaries between them. (C) Histogram of distribution of kmer-based tetranucleotide method (TETRA, %) values for all pairwise comparisons of the phylum *Spirochaetes*. The values are distributed in two main modes, with the first skewed to the right and with no clear boundaries between them. (D) POCP matrix of all pairwise comparisons of the phylum *Spirochaetes* that include a POCP value ≥ 45%. Values lower than the 45% threshold are coloured in white, all the other colours in the POCP matrix correspond to the continuous scale represented in the right.
**Figure S9. Annotation graph of all *Spirochaetes* species.** (A) Annotation graph of genome repartition by COG categories, including unknown function genes (COG category S). The genomes are ordered from left to right according to their phylogenetic position in Fig 1C, and COG categories are color-coded and ordered alphabetically as represented in the legend on the right. (B) Representation of the maximum annotation level repartition per genome, including unannotated genes (category S). The legend on the right illustrates the putative origin of the OGs per genome: B for bacterial, A for archaeal, V for viral and E for eukaryotic. The genomes are ordered from left to right according to their phylogenetic position in Fig 1C.
**Figure S10. Comparative analysis of the non-bacterial OGs in the main genera of *Spirochaetes*.** Percentage of OGs of putative eukaryotic (panel A), archaeal (panel B) or viral (panel C) origin in *Borreliaceae* spp. (grey dots), *Leptospira* spp. (blue dots), *Treponema* spp. (green dots) or *Brachyspira* spp. (purple dots). * p-value < 0.05, ** p-value < 0.01, *** p-value < 0.001, **** p-value < 0.0001. The data was analysed using a Brown-Forsythe and Welch ANOVA test with Dunnett’s T3 post-comparison test.
**Figure S11. Contribution of the individual variables to the Principal Component Analysis (PCA).** Percentage of contribution of each COG category to the PC1 and PC2 (panel A), PC1 only (panel B) and PC2 only (panel C). The red dashed line indicates the percentage of contribution if all variables contributed the same to the components (5%).
**Figure S12. Functional analysis of *Spirochaetes*.** Enrichment analysis of the COG categories in the non-spiral Spirochaetes (*B. porci*, *P. coccoides*, *S. pleomorpha*, *S. globosa* and *S. halotolerans*) versus all other *Spirochaetes* (panel A); or the P1+ *Leptospira* spp. versus all other *Leptospira* spp. (panel B). Log_2_FC values are arranged from lower to higher (left-to-right) and coloured according to the legend. The boxplots indicate the log_2_FC median and the first and third quartiles for all species within that group. (C) Principal Component Analysis (PCA) individuals plot of the COG category distribution within *Leptospira* spp., with the main subclades coloured as represented in the legend.
**Figure S13. OGs present in the Last Spirochaetal Common Ancestor** Presence/absence plot of the 511 OGs found to be present in the LSCA, with species in which the OG is present or absent marked in blue and grey, respectively. The Y axis contains the species ordered from top to bottom according to their phylogenetic position in Fig 1C. OGs are ordered by COG category and from most to least abundant. The colour-coded lower panel indicates the COG category according to the legend on the right.
**Figure S14. OGs lost by non-spiral *Spirochaetes*.** Distribution of the number of OGs found to be lost by the non-spiral *Spirochaetes* (*B. porci*, *P. coccoides*, *S. pleomorpha*, *S. globosa* and *S. halotolerans*) through the two approaches employed in the study (see Methods). OGs in the LOST type (95% conservation or higher in the closest clade spiral species) are coloured in dark blue while OGs in the ABSENT type (80% or higher conservation in all other spiral *Spirochaetes*) are coloured in light blue.
Table S1. Genomes used in this study and their main features.
Table S2. Percentage of conserved proteins (POCP) across all *Spirochaetes* species
Table S3. Annotation of the OGs of putative eukaryotic origin found in *Leptospira* spp.
Table S4. Relative percentage of the genome of each *Spirochaetes* species devoted to each COG functional category.
Table S5. Annotation of the OGs found present in the LSCA.
Table S6. OGs lost by the non-spiral *Spirochaetes*.
