# Supplementary material for: Linking genomic evolutionary transitions to ecological phenotypic adaptations in Spirochaetes
Source: bioRxiv. 2025 Jul 4:2025.07.04.663154. Preprint. [Version 1] doi: 10.1101/2025.07.04.663154 (PMC12236494; doi:10.1101/2025.07.04.663154)
Supplement: Supplement 1 — Figure S1. Pangenome analysis of the phylum Spirochaetes. (A) Pangenome accumulation plot of the Spirochaetes phylum representing the cumulative number of different OGs. This was calculated using 100 random iterations in the presence/absence matrix of OGs. Each blue dot represents one iteration, and the black line is the smooth curve of regression calculated using a generalized additive model (gam) with a cubic spline under the formula y ~ s(x, bs = “cs”). (B) Cumulative (green dots) and non-cumulative (blue dots) numbers of orthologs shared as the number of species increases in the range 2 to 172. The Y axis is represented in logarithmic scale to facilitate visualization. Figure S2. Phylogenetic comparisons of the Spirochaetes phylum (I). (A) Co-phylo plot representing the comparison between the phylogeny obtained under the unrooted homogeneous model of evolution (LG+F+I+R10, left side) and the unrooted heterogeneous model of evolution (LG+C20+R10, right side). Red lines connect the same leaves (species) in both trees. (B) Co-phylo plot representing the comparison between the phylogeny obtained under the rooted homogeneous model of evolution (LG+F+I+R10, left side) and the rooted heterogeneous model of evolution (LG+C20+R10, right side). Red lines connect the same leaves (species) in both trees. Figure S3. Phylogenetic comparisons of the Spirochaetes phylum (II). (A) Co-phylo plot representing the comparison between the phylogeny obtained under the rooted homogeneous model of evolution (LG+F+I+R10, left side) and the unrooted homogeneous model of evolution (LG+F+I+R10, right side). Red lines connect the same leaves (species) in both trees. (B) Co-phylo plot representing the comparison between the phylogeny obtained under the rooted heterogeneous model of evolution (LG+C20+R10, left side) and the unrooted heterogeneous model of evolution (LG+C20+R10, right side). Red lines connect the same leaves (species) in both trees. Figure S4. Phylogenetic comparisons of the Sp [file media-1.zip › supplementary data/SUPPLEMENTARY DATAb.docx]

**SUPPLEMENTARY FIGURES**

**Figure S1. Pangenome analysis of the phylum *Spirochaetes*.**

(A) Pangenome accumulation plot of the *Spirochaetes* phylum representing the cumulative number of different OGs. This was calculated using 100 random iterations in the presence/absence matrix of OGs. Each blue dot represents one iteration, and the black line is the smooth curve of regression calculated using a generalized additive model (gam) with a cubic spline under the formula y ~ s(x, bs = "cs"). (B) Cumulative (green dots) and non-cumulative (blue dots) numbers of orthologs shared as the number of species increases in the range 2 to 172. The Y axis is represented in logarithmic scale to facilitate visualization.

**Figure S2. Phylogenetic comparisons of the *Spirochaetes* phylum (I).**

(A) Co-phylo plot representing the comparison between the phylogeny obtained under the unrooted homogeneous model of evolution (LG+F+I+R10, left side) and the unrooted heterogeneous model of evolution (LG+C20+R10, right side). Red lines connect the same leaves (species) in both trees. (B) Co-phylo plot representing the comparison between the phylogeny obtained under the rooted homogeneous model of evolution (LG+F+I+R10, left side) and the rooted heterogeneous model of evolution (LG+C20+R10, right side). Red lines connect the same leaves (species) in both trees.

**Figure S3. Phylogenetic comparisons of the *Spirochaetes* phylum (II).**

(A) Co-phylo plot representing the comparison between the phylogeny obtained under the rooted homogeneous model of evolution (LG+F+I+R10, left side) and the unrooted homogeneous model of evolution (LG+F+I+R10, right side). Red lines connect the same leaves (species) in both trees. (B) Co-phylo plot representing the comparison between the phylogeny obtained under the rooted heterogeneous model of evolution (LG+C20+R10, left side) and the unrooted heterogeneous model of evolution (LG+C20+R10, right side). Red lines connect the same leaves (species) in both trees.

**Figure S4. Phylogenetic comparisons of the *Spirochaetes* phylum (III).**

(A) Co-phylo plot representing the comparison between the phylogeny obtained under the rooted heterogeneous model of evolution (LG+C20+R10, left side) and the rooted multi-species coalescence model of evolution (ASTRAL, right side). Red lines connect the same leaves (species) in both trees. (B) Presence/absence plot of the 140 soft-core genome markers (OGs) used for the phylogenetic inference, with species in which the OG is present marked in blue and absent in grey. The Y axis contains the species ordered from top to bottom according to their phylogenetic position in Fig 1C.

**Figure S5. An updated phylogeny for the *Treponematales* order.**

Phylogeny of the *Treponematales* order obtained under the model LG+F+I+R10 (best-fit bacterial model for *Spirochaetes*) using 440 soft-core genome markers and rooted using the closest clade in Fig 1C (*Sediminispirochaeta smaragdinae*, *Sediminispirochaeta bajacaliforniensis*, *Marispirochaeta aestuarii*). The two subclades within the *Treponema* genus are highlighted in blue (T1 subclade) and grey (T2 subclade). All nodes had SH-alrt and bootstrap support values of 100% except for the one splitting *T. parvum* from *T. socranskii*, *T. porcinum* and *T. bryantii*, which had 97/91% support.

**Figure S6. Average nucleotide identity matrix of the *Spirochaetes*.**

Phylogenetic tree of the cultivable species of *Spirochaetes*, with the main clades highlighted in colours (yellow for *Brachyspira* spp., dark grey for *Brevinematales*, cyan for *Leptospirales*, red for *Borreliaceae*, green for *Entomospira* spp., light blue for intermediate *Spirochaetia*, and dark blue for *Treponemataceae*), represented next to the pairwise matrix of Average Nucleotide Identity using the BLASTn method (ANIb, %). The colours in the ANIb matrix correspond to the continuous scale represented in the right. The intermediate coloured column represents the main clade classification.

**Figure S7. Kmer-based tetranucleotide matrix of the *Spirochaetes.***

Phylogenetic tree of the cultivable species of *Spirochaetes*, with the main clades highlighted in colours (yellow for *Brachyspira* spp., dark grey for *Brevinematales*, cyan for *Leptospirales*, red for *Borreliaceae*, green for *Entomospira* spp., light blue for intermediate *Spirochaetia*, and dark blue for *Treponemataceae*), represented next to the pairwise matrix of a kmer-based tetranucleotide method (TETRA, %). The colours in the TETRA matrix correspond to the continuous scale represented in the right. The intermediate coloured column represents the main clade classification.

**Figure S8. Percentage of conserved proteins as predictor of genus boundaries in *Spirochaetes*.**

(A) Histogram of distribution of Average Nucleotide Identity using the BLASTn method (ANIb, %) values for all pairwise comparisons of the phylum *Spirochaetes*. The distribution of the values is a single mode skewed uniformly to the right. (B) Histogram of distribution of Percentage of Conserved Proteins (POCP, %) values for all pairwise comparisons of the phylum *Spirochaetes*. The values are distributed in three main modes with clear boundaries between them. (C) Histogram of distribution of kmer-based tetranucleotide method (TETRA, %) values for all pairwise comparisons of the phylum *Spirochaetes*. The values are distributed in two main modes, with the first skewed to the right and with no clear boundaries between them. (D) POCP matrix of all pairwise comparisons of the phylum *Spirochaetes* that include a POCP value ≥ 45%. Values lower than the 45% threshold are coloured in white, all the other colours in the POCP matrix correspond to the continuous scale represented in the right.

**Figure S9. Annotation graph of all *Spirochaetes* species.**

(A) Annotation graph of genome repartition by COG categories, including unknown function genes (COG category S). The genomes are ordered from left to right according to their phylogenetic position in Fig 1C, and COG categories are color-coded and ordered alphabetically as represented in the legend on the right. (B) Representation of the maximum annotation level repartition per genome, including unannotated genes (category S). The legend on the right illustrates the putative origin of the OGs per genome: B for bacterial, A for archaeal, V for viral and E for eukaryotic. The genomes are ordered from left to right according to their phylogenetic position in Fig 1C.

**Figure S10. Comparative analysis of the non-bacterial OGs in the main genera of *Spirochaetes*.**

Percentage of OGs of putative eukaryotic (panel A), archaeal (panel B) or viral (panel C) origin in *Borreliaceae* spp. (grey dots), *Leptospira* spp. (blue dots), *Treponema* spp. (green dots) or *Brachyspira* spp. (purple dots). ***** p-value < 0.05, ****** p-value < 0.01, ******* p-value < 0.001, ******** p-value < 0.0001. The data was analysed using a Brown-Forsythe and Welch ANOVA test with Dunnett’s T3 post-comparison test.

**Figure S11. Contribution of the individual variables to the Principal Component Analysis (PCA).**

Percentage of contribution of each COG category to the PC1 and PC2 (panel A), PC1 only (panel B) and PC2 only (panel C). The red dashed line indicates the percentage of contribution if all variables contributed the same to the components (5%).

**Figure S12. Functional analysis of *Spirochaetes*.**

Enrichment analysis of the COG categories in the non-spiral Spirochaetes (*B. porci*, *P. coccoides*, *S. pleomorpha*, *S. globosa* and *S. halotolerans*) versus all other *Spirochaetes* (panel A); or the P1+ *Leptospira* spp. versus all other *Leptospira* spp. (panel B). Log_2_FC values are arranged from lower to higher (left-to-right) and coloured according to the legend. The boxplots indicate the log_2_FC median and the first and third quartiles for all species within that group. (C) Principal Component Analysis (PCA) individuals plot of the COG category distribution within *Leptospira* spp., with the main subclades coloured as represented in the legend.

**Figure S13. OGs present in the Last Spirochaetal Common Ancestor**

Presence/absence plot of the 511 OGs found to be present in the LSCA, with species in which the OG is present or absent marked in blue and grey, respectively. The Y axis contains the species ordered from top to bottom according to their phylogenetic position in Fig 1C. OGs are ordered by COG category and from most to least abundant. The colour-coded lower panel indicates the COG category according to the legend on the right.

**Figure S14. OGs lost by non-spiral *Spirochaetes*.**

Distribution of the number of OGs found to be lost by the non-spiral *Spirochaetes* (*B. porci*, *P. coccoides*, *S. pleomorpha*, *S. globosa* and *S. halotolerans*) through the two approaches employed in the study (see Methods). OGs in the LOST type (95% conservation or higher in the closest clade spiral species) are coloured in dark blue while OGs in the ABSENT type (80% or higher conservation in all other spiral *Spirochaetes*) are coloured in light blue.

**SUPPLEMENTARY TABLES**

**Table S1. Genomes used in this study and their main features.**

**Table S2. Percentage of conserved proteins (POCP) across all *Spirochaetes* species**

**Table S3. Annotation of the OGs of putative eukaryotic origin found in *Leptospira* spp.**

**Table S4. Relative percentage of the genome of each *Spirochaetes* species devoted to each COG functional category.**

**Table S5. Annotation of the OGs found present in the LSCA.**

**Table S6. OGs lost by the non-spiral *Spirochaetes*.**
