# Supplementary figures and images for: Linking genomic evolutionary transitions to ecological phenotypic adaptations in Spirochaetes
Source: bioRxiv. 2025 Jul 4:2025.07.04.663154. Preprint. [Version 1] doi: 10.1101/2025.07.04.663154 (PMC12236494; doi:10.1101/2025.07.04.663154)

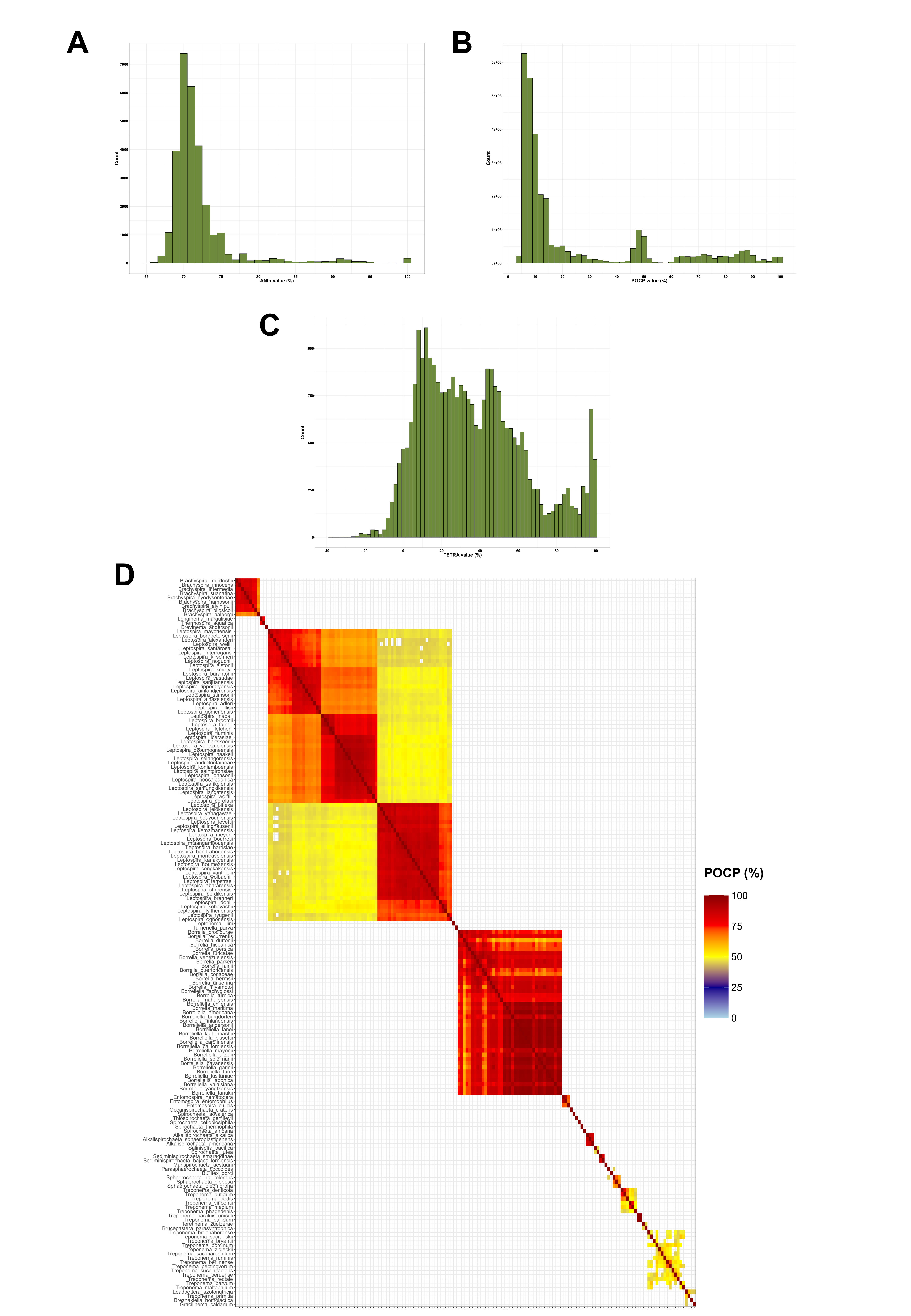

Supplement: Supplement 1 — Figure S1. Pangenome analysis of the phylum Spirochaetes. (A) Pangenome accumulation plot of the Spirochaetes phylum representing the cumulative number of different OGs. This was calculated using 100 random iterations in the presence/absence matrix of OGs. Each blue dot represents one iteration, and the black line is the smooth curve of regression calculated using a generalized additive model (gam) with a cubic spline under the formula y ~ s(x, bs = “cs”). (B) Cumulative (green dots) and non-cumulative (blue dots) numbers of orthologs shared as the number of species increases in the range 2 to 172. The Y axis is represented in logarithmic scale to facilitate visualization. Figure S2. Phylogenetic comparisons of the Spirochaetes phylum (I). (A) Co-phylo plot representing the comparison between the phylogeny obtained under the unrooted homogeneous model of evolution (LG+F+I+R10, left side) and the unrooted heterogeneous model of evolution (LG+C20+R10, right side). Red lines connect the same leaves (species) in both trees. (B) Co-phylo plot representing the comparison between the phylogeny obtained under the rooted homogeneous model of evolution (LG+F+I+R10, left side) and the rooted heterogeneous model of evolution (LG+C20+R10, right side). Red lines connect the same leaves (species) in both trees. Figure S3. Phylogenetic comparisons of the Spirochaetes phylum (II). (A) Co-phylo plot representing the comparison between the phylogeny obtained under the rooted homogeneous model of evolution (LG+F+I+R10, left side) and the unrooted homogeneous model of evolution (LG+F+I+R10, right side). Red lines connect the same leaves (species) in both trees. (B) Co-phylo plot representing the comparison between the phylogeny obtained under the rooted heterogeneous model of evolution (LG+C20+R10, left side) and the unrooted heterogeneous model of evolution (LG+C20+R10, right side). Red lines connect the same leaves (species) in both trees. Figure S4. Phylogenetic comparisons of the Sp [file media-1.zip › supplementary data/Figure_S8.png]

**A**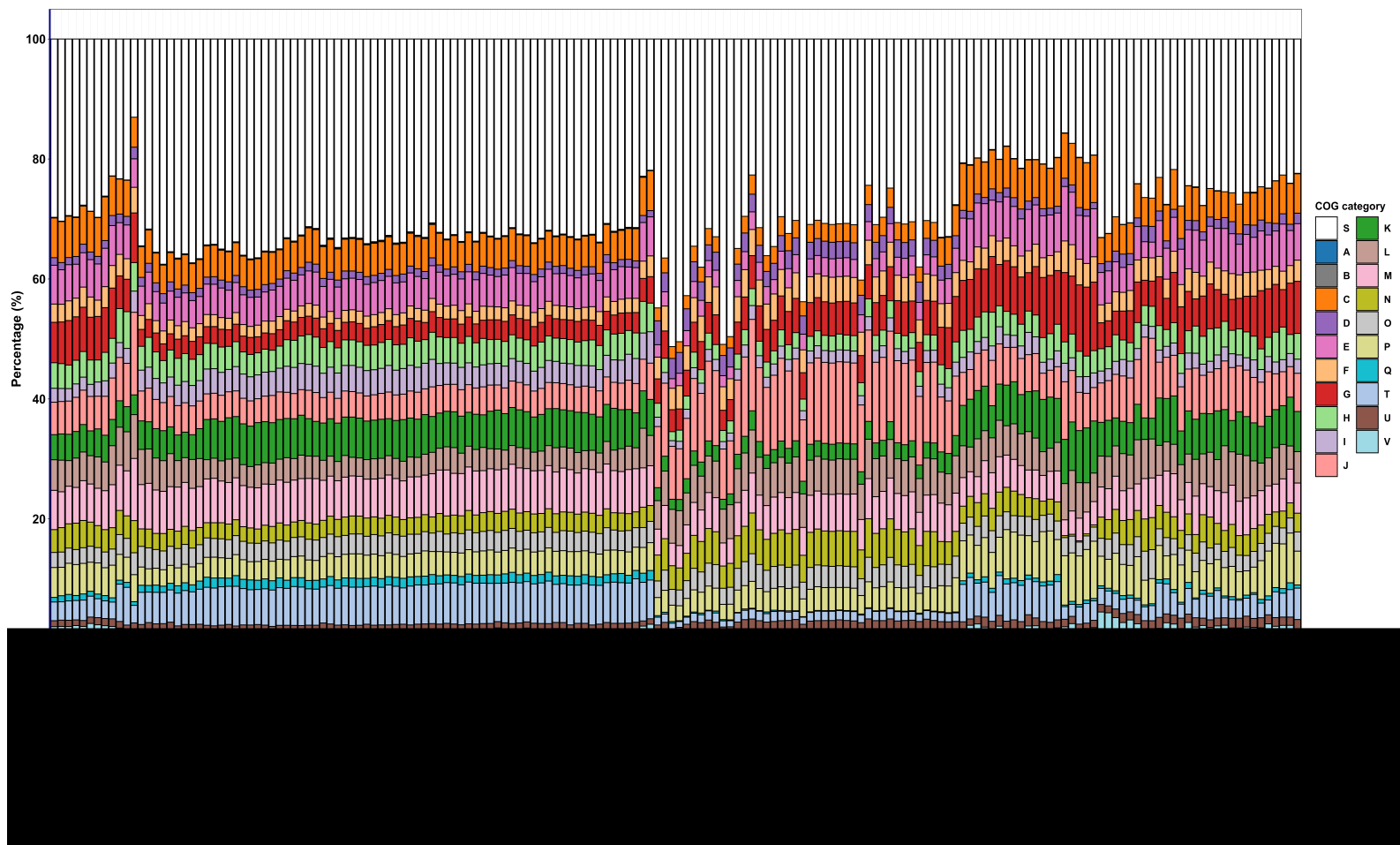**B**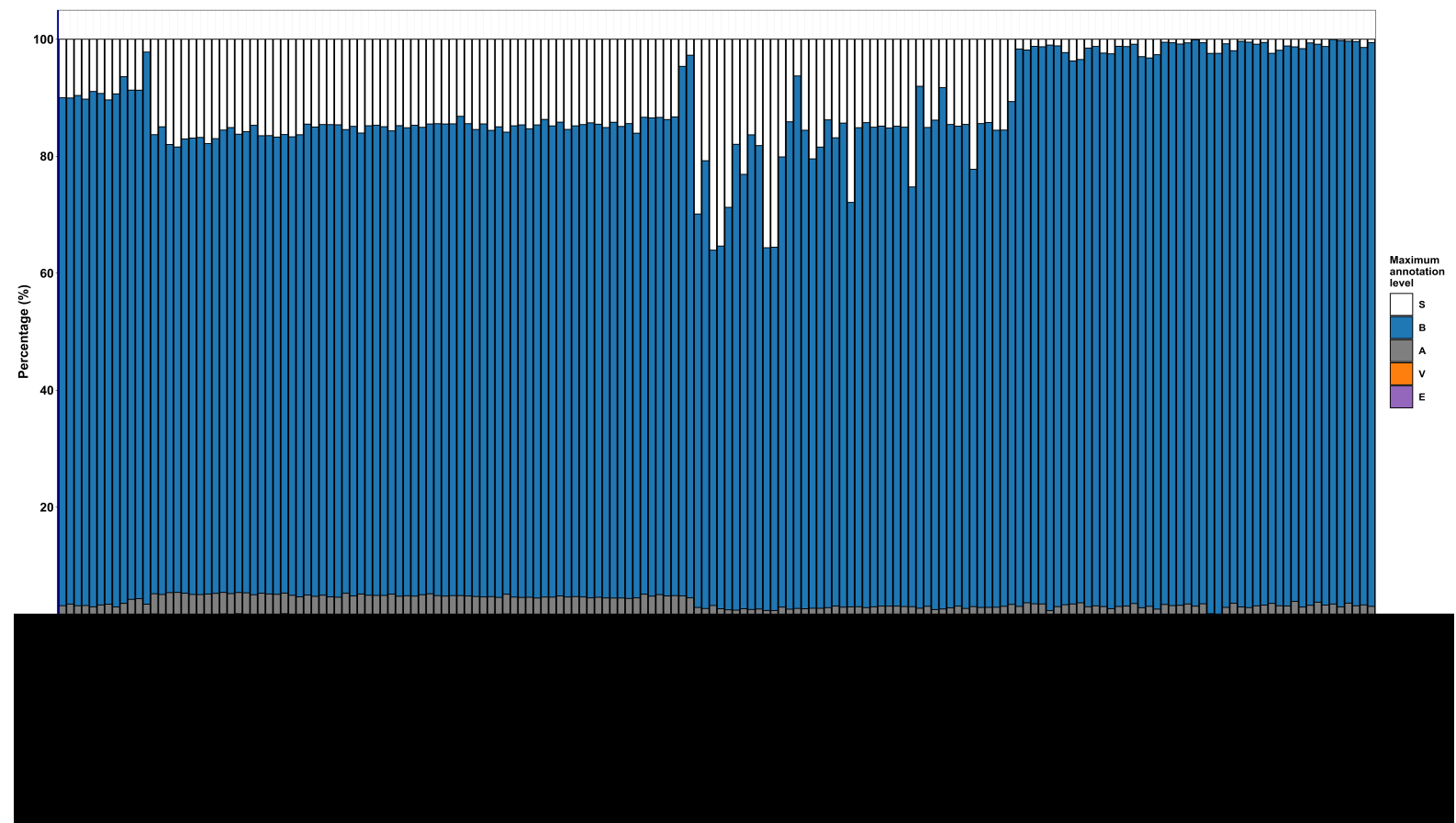

Supplement: Supplement 1 — Figure S1. Pangenome analysis of the phylum Spirochaetes. (A) Pangenome accumulation plot of the Spirochaetes phylum representing the cumulative number of different OGs. This was calculated using 100 random iterations in the presence/absence matrix of OGs. Each blue dot represents one iteration, and the black line is the smooth curve of regression calculated using a generalized additive model (gam) with a cubic spline under the formula y ~ s(x, bs = “cs”). (B) Cumulative (green dots) and non-cumulative (blue dots) numbers of orthologs shared as the number of species increases in the range 2 to 172. The Y axis is represented in logarithmic scale to facilitate visualization. Figure S2. Phylogenetic comparisons of the Spirochaetes phylum (I). (A) Co-phylo plot representing the comparison between the phylogeny obtained under the unrooted homogeneous model of evolution (LG+F+I+R10, left side) and the unrooted heterogeneous model of evolution (LG+C20+R10, right side). Red lines connect the same leaves (species) in both trees. (B) Co-phylo plot representing the comparison between the phylogeny obtained under the rooted homogeneous model of evolution (LG+F+I+R10, left side) and the rooted heterogeneous model of evolution (LG+C20+R10, right side). Red lines connect the same leaves (species) in both trees. Figure S3. Phylogenetic comparisons of the Spirochaetes phylum (II). (A) Co-phylo plot representing the comparison between the phylogeny obtained under the rooted homogeneous model of evolution (LG+F+I+R10, left side) and the unrooted homogeneous model of evolution (LG+F+I+R10, right side). Red lines connect the same leaves (species) in both trees. (B) Co-phylo plot representing the comparison between the phylogeny obtained under the rooted heterogeneous model of evolution (LG+C20+R10, left side) and the unrooted heterogeneous model of evolution (LG+C20+R10, right side). Red lines connect the same leaves (species) in both trees. Figure S4. Phylogenetic comparisons of the Sp [file media-1.zip › supplementary data/Figure_S9.pdf]

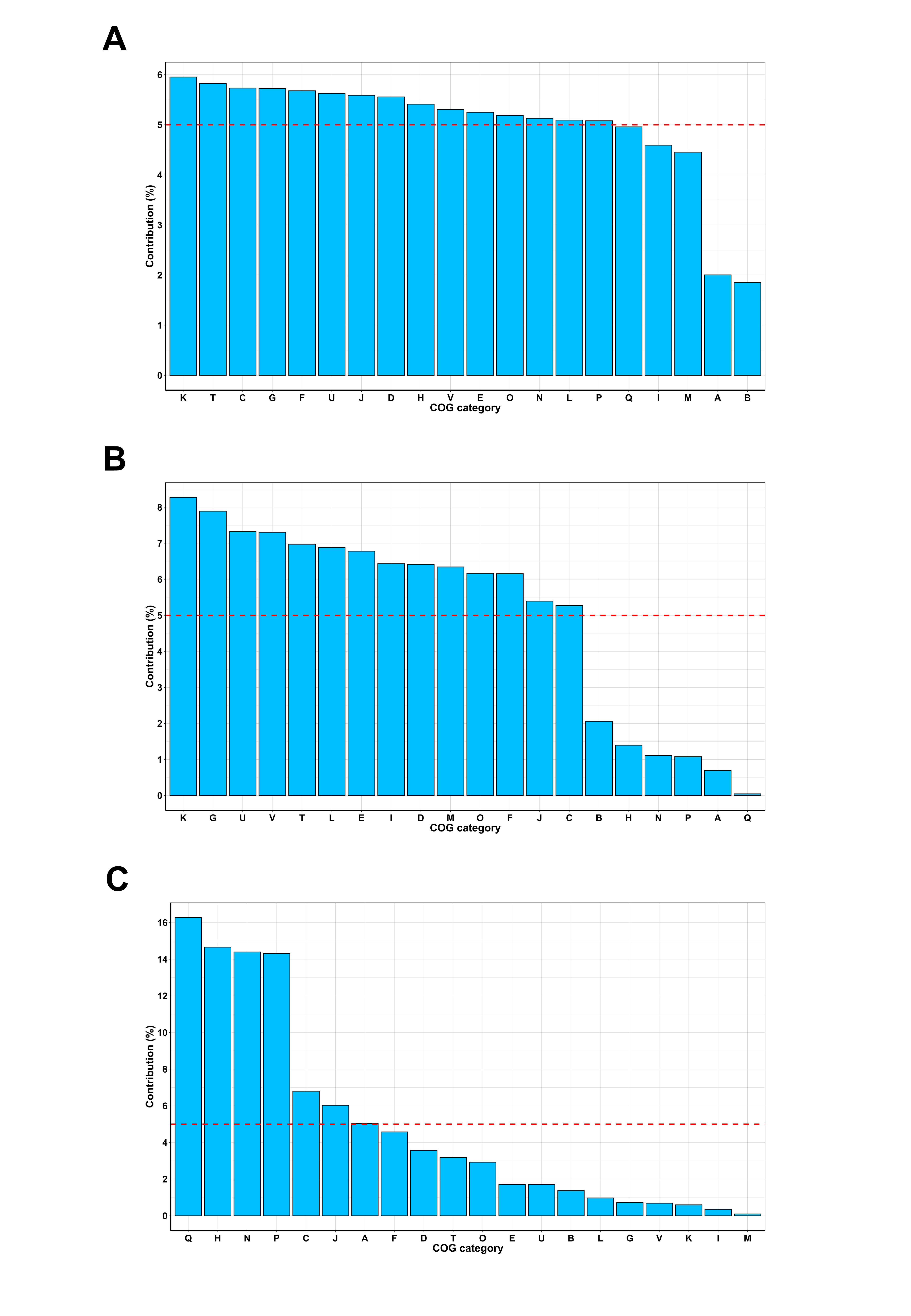

Supplement: Supplement 1 — Figure S1. Pangenome analysis of the phylum Spirochaetes. (A) Pangenome accumulation plot of the Spirochaetes phylum representing the cumulative number of different OGs. This was calculated using 100 random iterations in the presence/absence matrix of OGs. Each blue dot represents one iteration, and the black line is the smooth curve of regression calculated using a generalized additive model (gam) with a cubic spline under the formula y ~ s(x, bs = “cs”). (B) Cumulative (green dots) and non-cumulative (blue dots) numbers of orthologs shared as the number of species increases in the range 2 to 172. The Y axis is represented in logarithmic scale to facilitate visualization. Figure S2. Phylogenetic comparisons of the Spirochaetes phylum (I). (A) Co-phylo plot representing the comparison between the phylogeny obtained under the unrooted homogeneous model of evolution (LG+F+I+R10, left side) and the unrooted heterogeneous model of evolution (LG+C20+R10, right side). Red lines connect the same leaves (species) in both trees. (B) Co-phylo plot representing the comparison between the phylogeny obtained under the rooted homogeneous model of evolution (LG+F+I+R10, left side) and the rooted heterogeneous model of evolution (LG+C20+R10, right side). Red lines connect the same leaves (species) in both trees. Figure S3. Phylogenetic comparisons of the Spirochaetes phylum (II). (A) Co-phylo plot representing the comparison between the phylogeny obtained under the rooted homogeneous model of evolution (LG+F+I+R10, left side) and the unrooted homogeneous model of evolution (LG+F+I+R10, right side). Red lines connect the same leaves (species) in both trees. (B) Co-phylo plot representing the comparison between the phylogeny obtained under the rooted heterogeneous model of evolution (LG+C20+R10, left side) and the unrooted heterogeneous model of evolution (LG+C20+R10, right side). Red lines connect the same leaves (species) in both trees. Figure S4. Phylogenetic comparisons of the Sp [file media-1.zip › supplementary data/Figure_S11.png]

**A**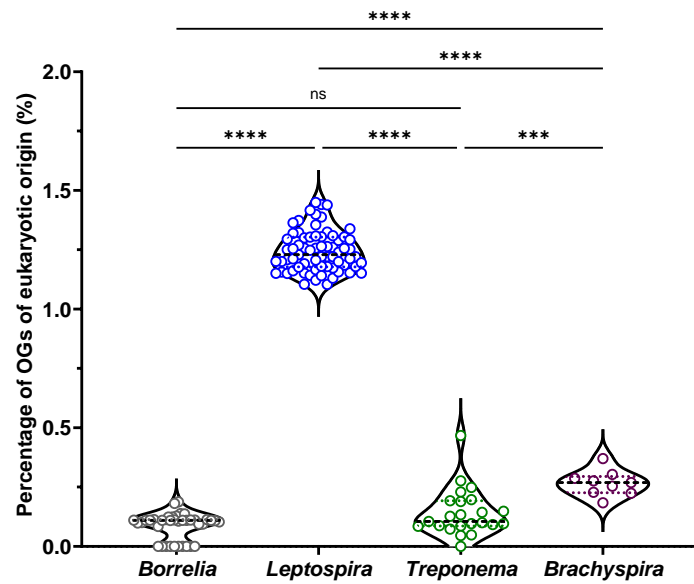**B**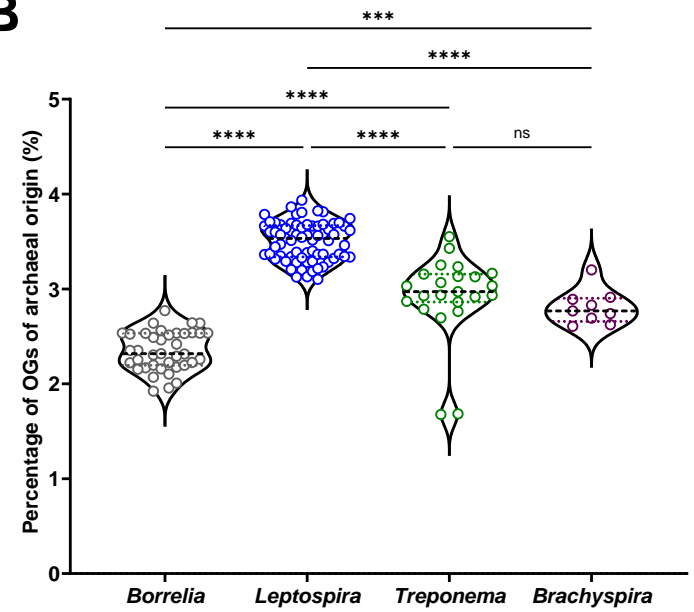**C**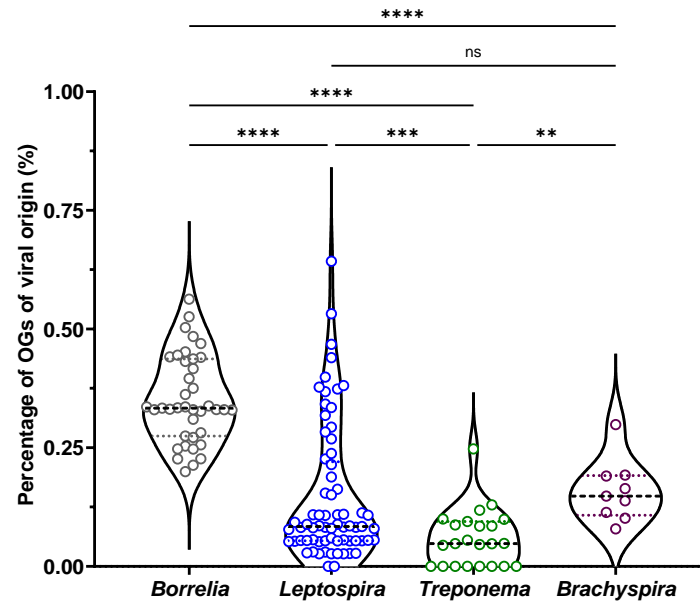

Supplement: Supplement 1 — Figure S1. Pangenome analysis of the phylum Spirochaetes. (A) Pangenome accumulation plot of the Spirochaetes phylum representing the cumulative number of different OGs. This was calculated using 100 random iterations in the presence/absence matrix of OGs. Each blue dot represents one iteration, and the black line is the smooth curve of regression calculated using a generalized additive model (gam) with a cubic spline under the formula y ~ s(x, bs = “cs”). (B) Cumulative (green dots) and non-cumulative (blue dots) numbers of orthologs shared as the number of species increases in the range 2 to 172. The Y axis is represented in logarithmic scale to facilitate visualization. Figure S2. Phylogenetic comparisons of the Spirochaetes phylum (I). (A) Co-phylo plot representing the comparison between the phylogeny obtained under the unrooted homogeneous model of evolution (LG+F+I+R10, left side) and the unrooted heterogeneous model of evolution (LG+C20+R10, right side). Red lines connect the same leaves (species) in both trees. (B) Co-phylo plot representing the comparison between the phylogeny obtained under the rooted homogeneous model of evolution (LG+F+I+R10, left side) and the rooted heterogeneous model of evolution (LG+C20+R10, right side). Red lines connect the same leaves (species) in both trees. Figure S3. Phylogenetic comparisons of the Spirochaetes phylum (II). (A) Co-phylo plot representing the comparison between the phylogeny obtained under the rooted homogeneous model of evolution (LG+F+I+R10, left side) and the unrooted homogeneous model of evolution (LG+F+I+R10, right side). Red lines connect the same leaves (species) in both trees. (B) Co-phylo plot representing the comparison between the phylogeny obtained under the rooted heterogeneous model of evolution (LG+C20+R10, left side) and the unrooted heterogeneous model of evolution (LG+C20+R10, right side). Red lines connect the same leaves (species) in both trees. Figure S4. Phylogenetic comparisons of the Sp [file media-1.zip › supplementary data/Figure_S10.pdf]

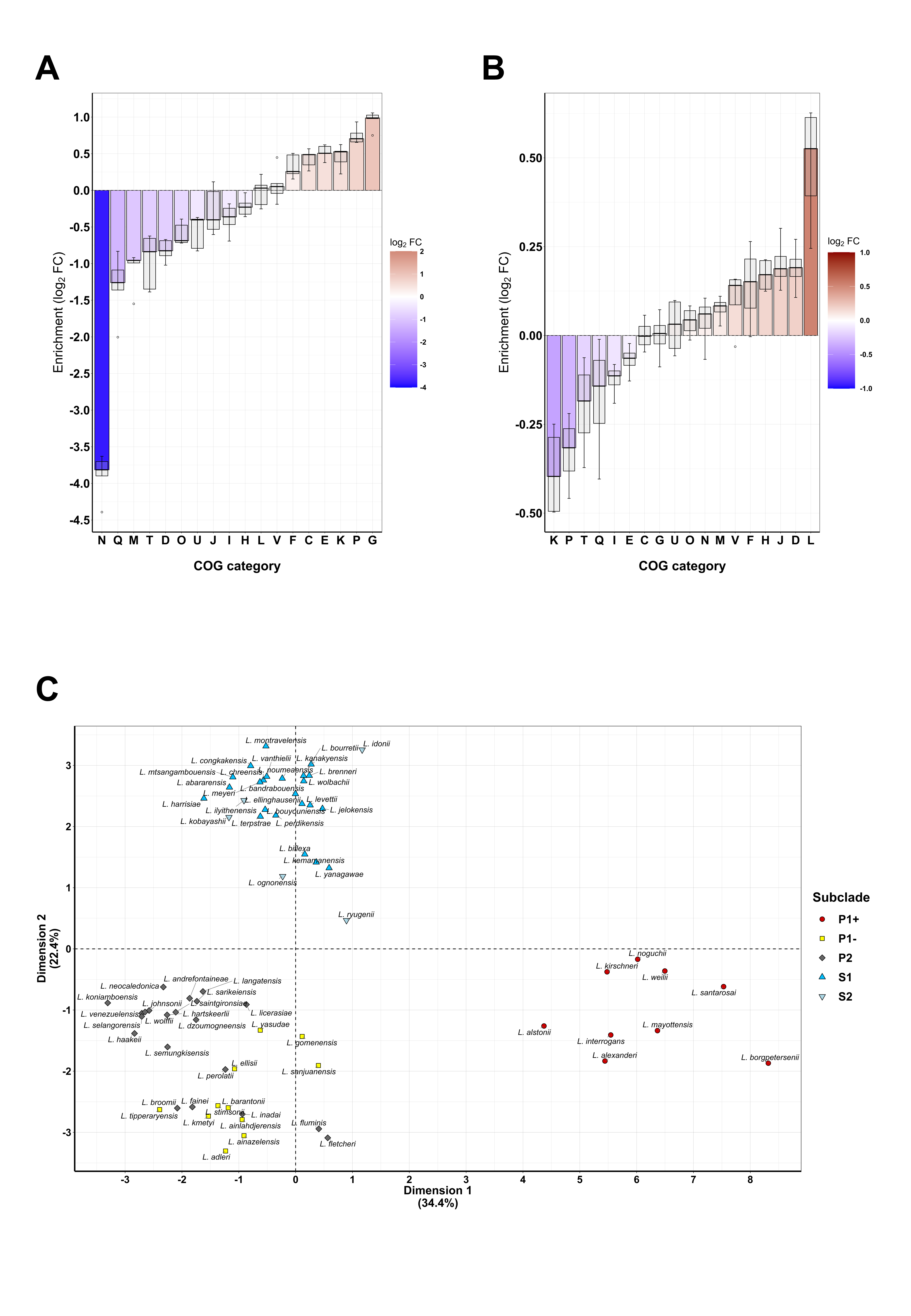

Supplement: Supplement 1 — Figure S1. Pangenome analysis of the phylum Spirochaetes. (A) Pangenome accumulation plot of the Spirochaetes phylum representing the cumulative number of different OGs. This was calculated using 100 random iterations in the presence/absence matrix of OGs. Each blue dot represents one iteration, and the black line is the smooth curve of regression calculated using a generalized additive model (gam) with a cubic spline under the formula y ~ s(x, bs = “cs”). (B) Cumulative (green dots) and non-cumulative (blue dots) numbers of orthologs shared as the number of species increases in the range 2 to 172. The Y axis is represented in logarithmic scale to facilitate visualization. Figure S2. Phylogenetic comparisons of the Spirochaetes phylum (I). (A) Co-phylo plot representing the comparison between the phylogeny obtained under the unrooted homogeneous model of evolution (LG+F+I+R10, left side) and the unrooted heterogeneous model of evolution (LG+C20+R10, right side). Red lines connect the same leaves (species) in both trees. (B) Co-phylo plot representing the comparison between the phylogeny obtained under the rooted homogeneous model of evolution (LG+F+I+R10, left side) and the rooted heterogeneous model of evolution (LG+C20+R10, right side). Red lines connect the same leaves (species) in both trees. Figure S3. Phylogenetic comparisons of the Spirochaetes phylum (II). (A) Co-phylo plot representing the comparison between the phylogeny obtained under the rooted homogeneous model of evolution (LG+F+I+R10, left side) and the unrooted homogeneous model of evolution (LG+F+I+R10, right side). Red lines connect the same leaves (species) in both trees. (B) Co-phylo plot representing the comparison between the phylogeny obtained under the rooted heterogeneous model of evolution (LG+C20+R10, left side) and the unrooted heterogeneous model of evolution (LG+C20+R10, right side). Red lines connect the same leaves (species) in both trees. Figure S4. Phylogenetic comparisons of the Sp [file media-1.zip › supplementary data/Figure_S12.png]

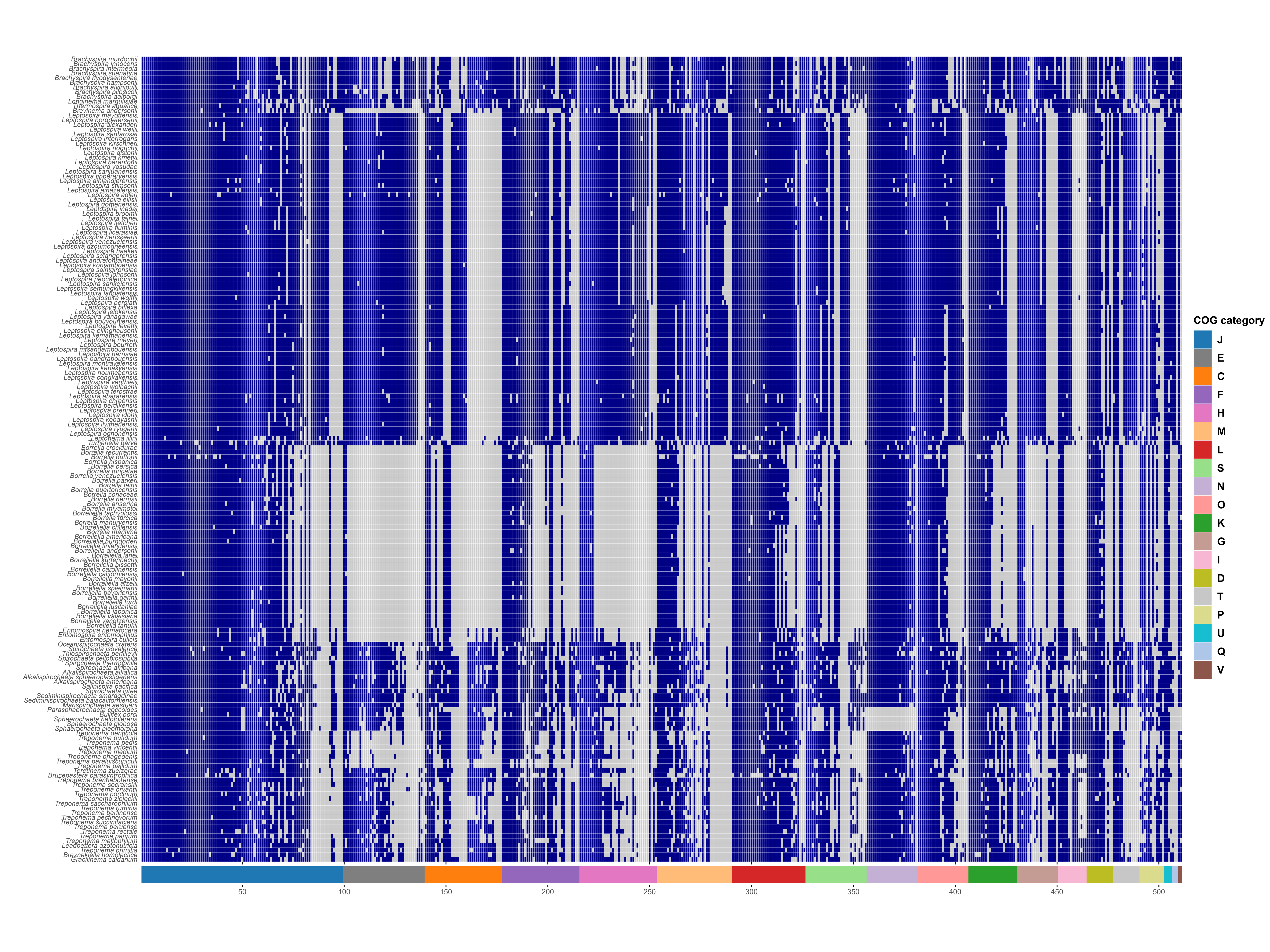

Supplement: Supplement 1 — Figure S1. Pangenome analysis of the phylum Spirochaetes. (A) Pangenome accumulation plot of the Spirochaetes phylum representing the cumulative number of different OGs. This was calculated using 100 random iterations in the presence/absence matrix of OGs. Each blue dot represents one iteration, and the black line is the smooth curve of regression calculated using a generalized additive model (gam) with a cubic spline under the formula y ~ s(x, bs = “cs”). (B) Cumulative (green dots) and non-cumulative (blue dots) numbers of orthologs shared as the number of species increases in the range 2 to 172. The Y axis is represented in logarithmic scale to facilitate visualization. Figure S2. Phylogenetic comparisons of the Spirochaetes phylum (I). (A) Co-phylo plot representing the comparison between the phylogeny obtained under the unrooted homogeneous model of evolution (LG+F+I+R10, left side) and the unrooted heterogeneous model of evolution (LG+C20+R10, right side). Red lines connect the same leaves (species) in both trees. (B) Co-phylo plot representing the comparison between the phylogeny obtained under the rooted homogeneous model of evolution (LG+F+I+R10, left side) and the rooted heterogeneous model of evolution (LG+C20+R10, right side). Red lines connect the same leaves (species) in both trees. Figure S3. Phylogenetic comparisons of the Spirochaetes phylum (II). (A) Co-phylo plot representing the comparison between the phylogeny obtained under the rooted homogeneous model of evolution (LG+F+I+R10, left side) and the unrooted homogeneous model of evolution (LG+F+I+R10, right side). Red lines connect the same leaves (species) in both trees. (B) Co-phylo plot representing the comparison between the phylogeny obtained under the rooted heterogeneous model of evolution (LG+C20+R10, left side) and the unrooted heterogeneous model of evolution (LG+C20+R10, right side). Red lines connect the same leaves (species) in both trees. Figure S4. Phylogenetic comparisons of the Sp [file media-1.zip › supplementary data/Figure_S13.png]

**A**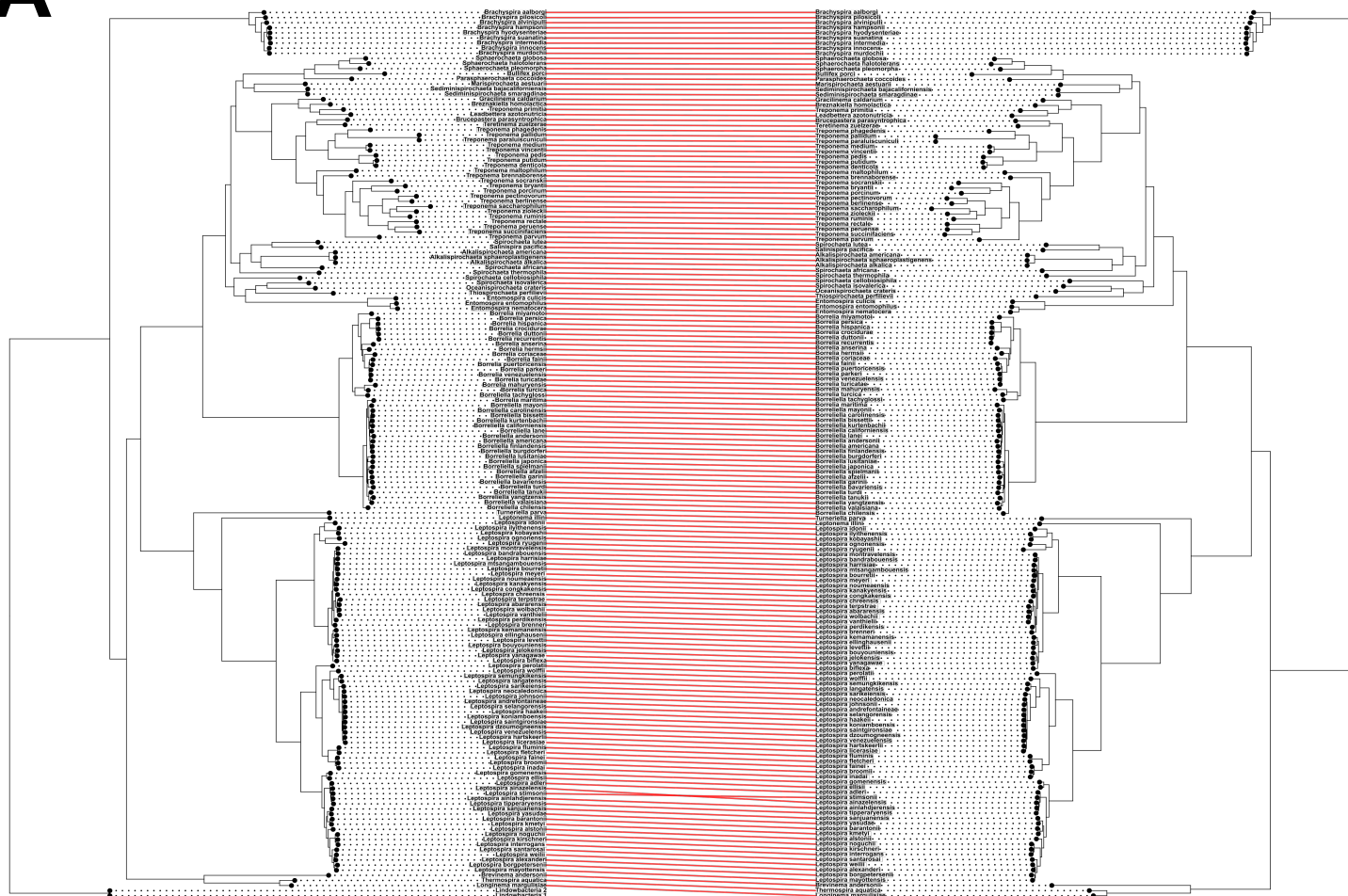**B**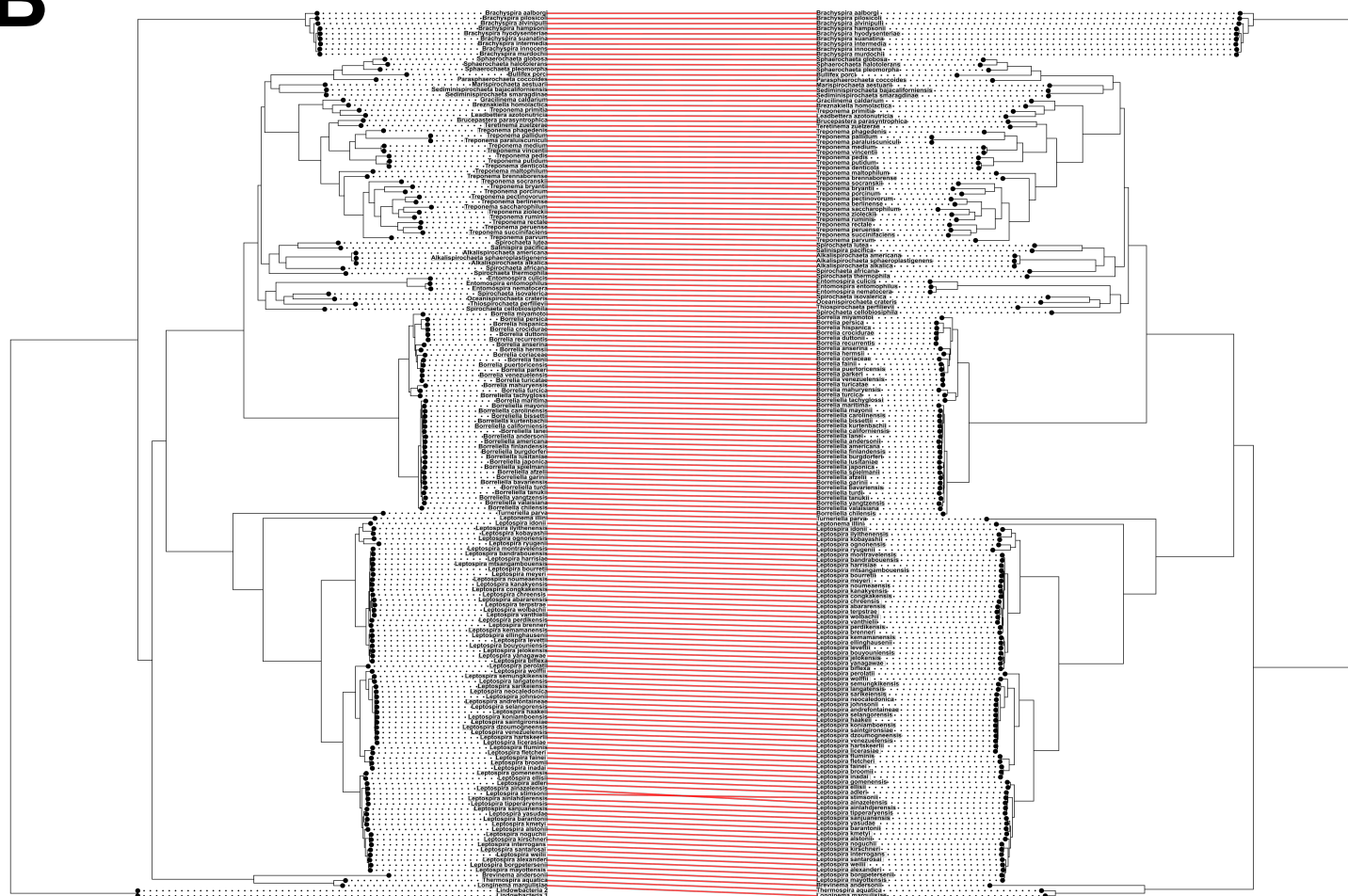

Supplement: Supplement 1 — Figure S1. Pangenome analysis of the phylum Spirochaetes. (A) Pangenome accumulation plot of the Spirochaetes phylum representing the cumulative number of different OGs. This was calculated using 100 random iterations in the presence/absence matrix of OGs. Each blue dot represents one iteration, and the black line is the smooth curve of regression calculated using a generalized additive model (gam) with a cubic spline under the formula y ~ s(x, bs = “cs”). (B) Cumulative (green dots) and non-cumulative (blue dots) numbers of orthologs shared as the number of species increases in the range 2 to 172. The Y axis is represented in logarithmic scale to facilitate visualization. Figure S2. Phylogenetic comparisons of the Spirochaetes phylum (I). (A) Co-phylo plot representing the comparison between the phylogeny obtained under the unrooted homogeneous model of evolution (LG+F+I+R10, left side) and the unrooted heterogeneous model of evolution (LG+C20+R10, right side). Red lines connect the same leaves (species) in both trees. (B) Co-phylo plot representing the comparison between the phylogeny obtained under the rooted homogeneous model of evolution (LG+F+I+R10, left side) and the rooted heterogeneous model of evolution (LG+C20+R10, right side). Red lines connect the same leaves (species) in both trees. Figure S3. Phylogenetic comparisons of the Spirochaetes phylum (II). (A) Co-phylo plot representing the comparison between the phylogeny obtained under the rooted homogeneous model of evolution (LG+F+I+R10, left side) and the unrooted homogeneous model of evolution (LG+F+I+R10, right side). Red lines connect the same leaves (species) in both trees. (B) Co-phylo plot representing the comparison between the phylogeny obtained under the rooted heterogeneous model of evolution (LG+C20+R10, left side) and the unrooted heterogeneous model of evolution (LG+C20+R10, right side). Red lines connect the same leaves (species) in both trees. Figure S4. Phylogenetic comparisons of the Sp [file media-1.zip › supplementary data/Figure_S3.pdf]

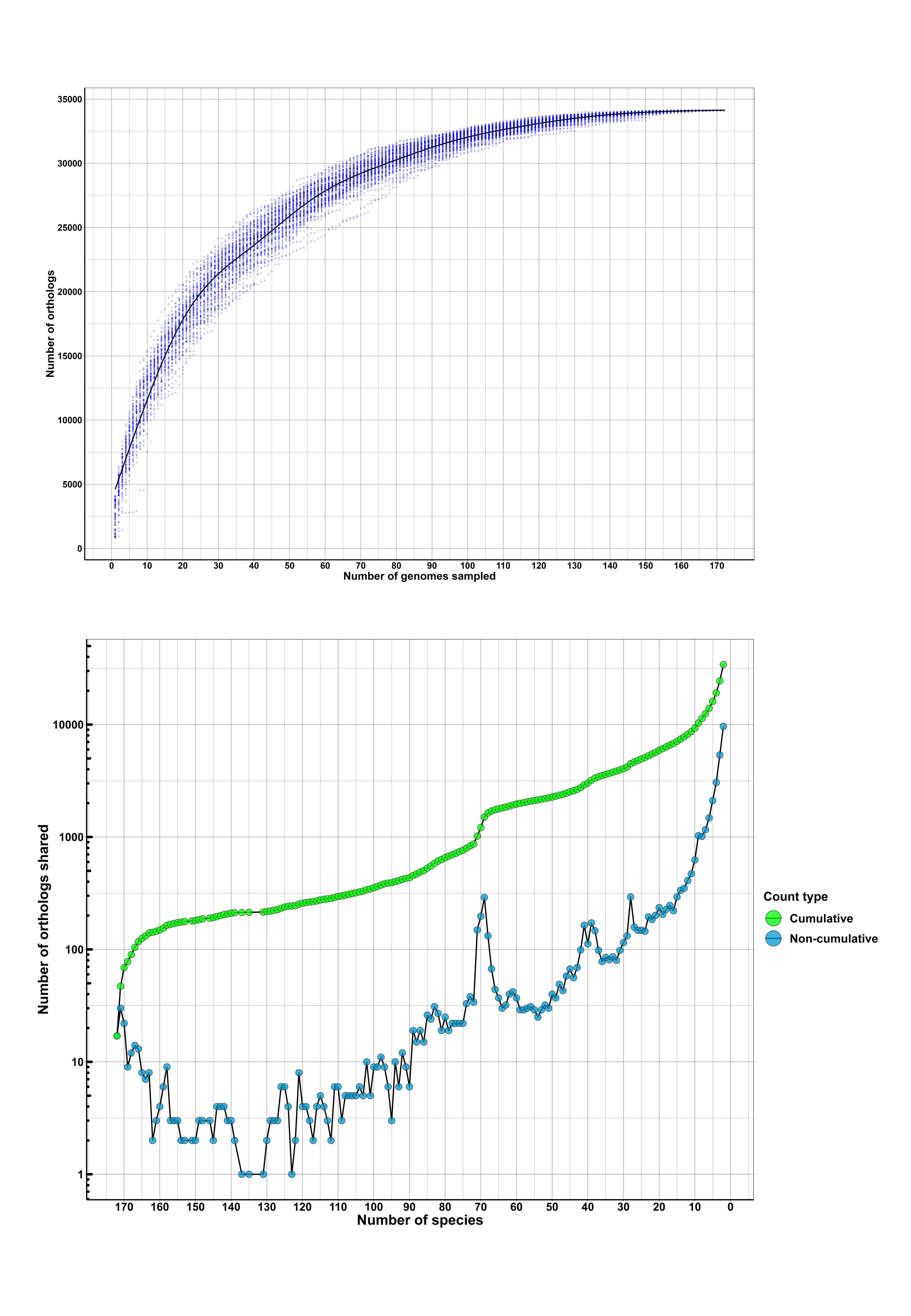

Supplement: Supplement 1 — Figure S1. Pangenome analysis of the phylum Spirochaetes. (A) Pangenome accumulation plot of the Spirochaetes phylum representing the cumulative number of different OGs. This was calculated using 100 random iterations in the presence/absence matrix of OGs. Each blue dot represents one iteration, and the black line is the smooth curve of regression calculated using a generalized additive model (gam) with a cubic spline under the formula y ~ s(x, bs = “cs”). (B) Cumulative (green dots) and non-cumulative (blue dots) numbers of orthologs shared as the number of species increases in the range 2 to 172. The Y axis is represented in logarithmic scale to facilitate visualization. Figure S2. Phylogenetic comparisons of the Spirochaetes phylum (I). (A) Co-phylo plot representing the comparison between the phylogeny obtained under the unrooted homogeneous model of evolution (LG+F+I+R10, left side) and the unrooted heterogeneous model of evolution (LG+C20+R10, right side). Red lines connect the same leaves (species) in both trees. (B) Co-phylo plot representing the comparison between the phylogeny obtained under the rooted homogeneous model of evolution (LG+F+I+R10, left side) and the rooted heterogeneous model of evolution (LG+C20+R10, right side). Red lines connect the same leaves (species) in both trees. Figure S3. Phylogenetic comparisons of the Spirochaetes phylum (II). (A) Co-phylo plot representing the comparison between the phylogeny obtained under the rooted homogeneous model of evolution (LG+F+I+R10, left side) and the unrooted homogeneous model of evolution (LG+F+I+R10, right side). Red lines connect the same leaves (species) in both trees. (B) Co-phylo plot representing the comparison between the phylogeny obtained under the rooted heterogeneous model of evolution (LG+C20+R10, left side) and the unrooted heterogeneous model of evolution (LG+C20+R10, right side). Red lines connect the same leaves (species) in both trees. Figure S4. Phylogenetic comparisons of the Sp [file media-1.zip › supplementary data/Figure_S1.png]

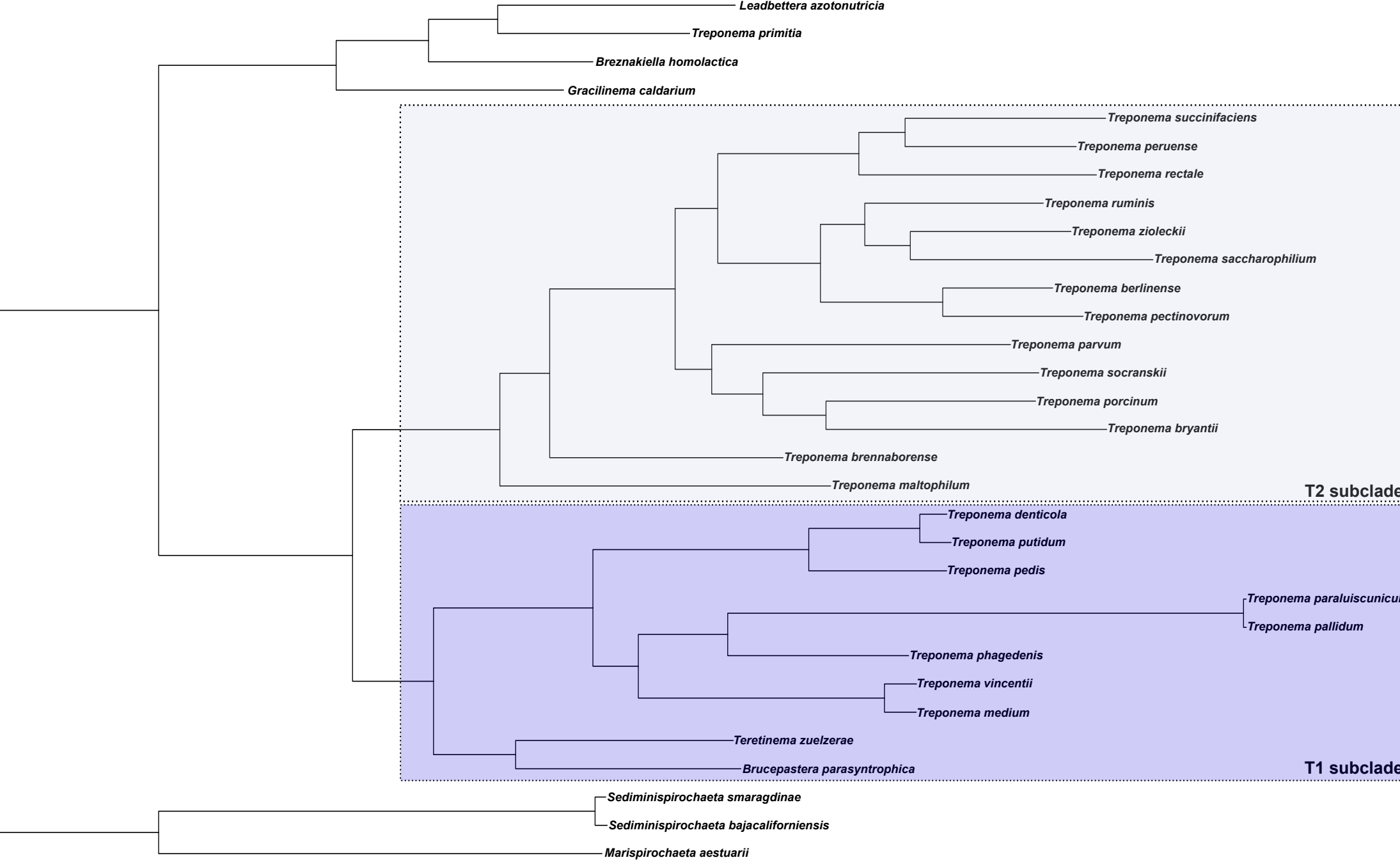

0.2

Supplement: Supplement 1 — Figure S1. Pangenome analysis of the phylum Spirochaetes. (A) Pangenome accumulation plot of the Spirochaetes phylum representing the cumulative number of different OGs. This was calculated using 100 random iterations in the presence/absence matrix of OGs. Each blue dot represents one iteration, and the black line is the smooth curve of regression calculated using a generalized additive model (gam) with a cubic spline under the formula y ~ s(x, bs = “cs”). (B) Cumulative (green dots) and non-cumulative (blue dots) numbers of orthologs shared as the number of species increases in the range 2 to 172. The Y axis is represented in logarithmic scale to facilitate visualization. Figure S2. Phylogenetic comparisons of the Spirochaetes phylum (I). (A) Co-phylo plot representing the comparison between the phylogeny obtained under the unrooted homogeneous model of evolution (LG+F+I+R10, left side) and the unrooted heterogeneous model of evolution (LG+C20+R10, right side). Red lines connect the same leaves (species) in both trees. (B) Co-phylo plot representing the comparison between the phylogeny obtained under the rooted homogeneous model of evolution (LG+F+I+R10, left side) and the rooted heterogeneous model of evolution (LG+C20+R10, right side). Red lines connect the same leaves (species) in both trees. Figure S3. Phylogenetic comparisons of the Spirochaetes phylum (II). (A) Co-phylo plot representing the comparison between the phylogeny obtained under the rooted homogeneous model of evolution (LG+F+I+R10, left side) and the unrooted homogeneous model of evolution (LG+F+I+R10, right side). Red lines connect the same leaves (species) in both trees. (B) Co-phylo plot representing the comparison between the phylogeny obtained under the rooted heterogeneous model of evolution (LG+C20+R10, left side) and the unrooted heterogeneous model of evolution (LG+C20+R10, right side). Red lines connect the same leaves (species) in both trees. Figure S4. Phylogenetic comparisons of the Sp [file media-1.zip › supplementary data/Figure_S5.pdf]

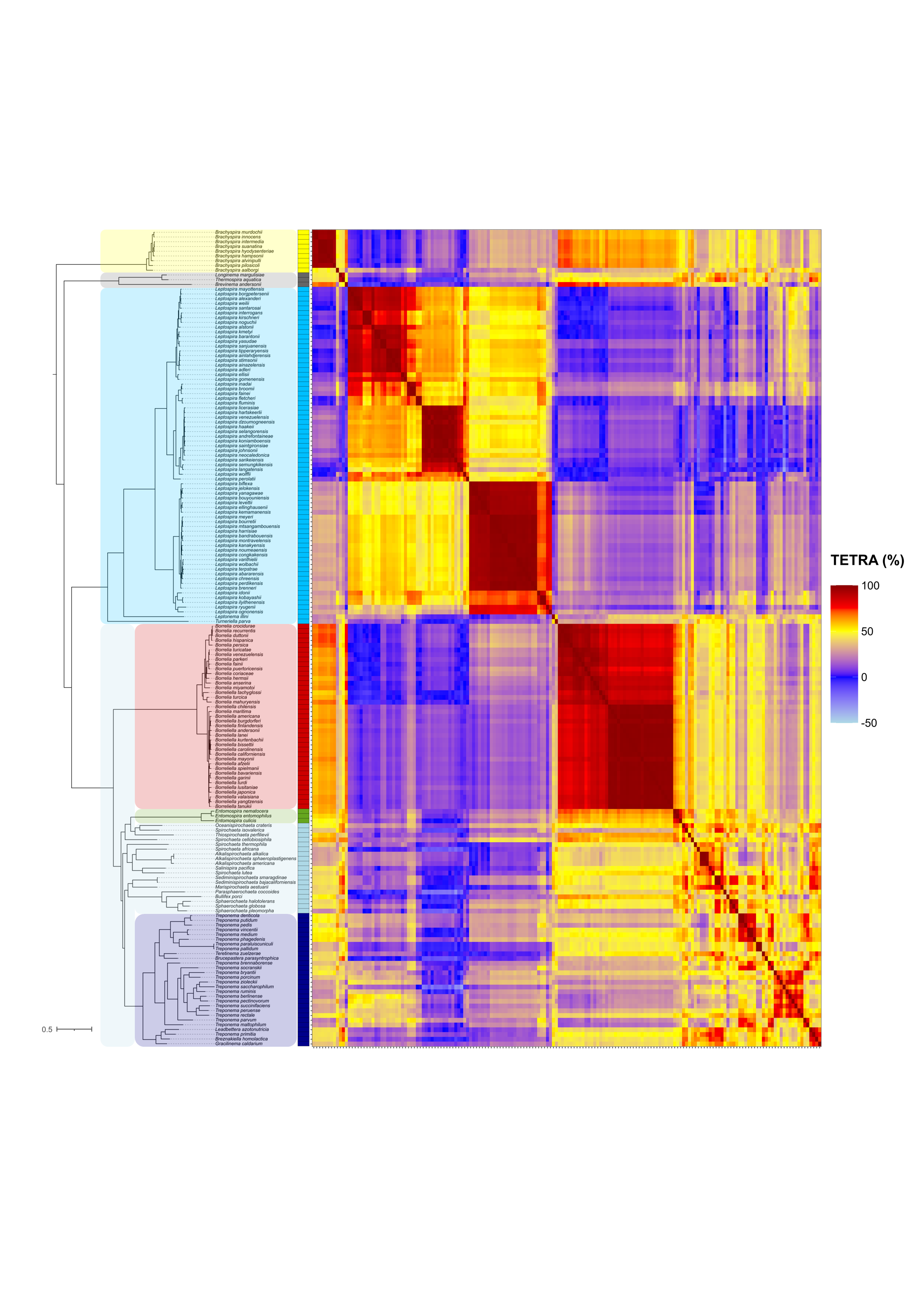

Supplement: Supplement 1 — Figure S1. Pangenome analysis of the phylum Spirochaetes. (A) Pangenome accumulation plot of the Spirochaetes phylum representing the cumulative number of different OGs. This was calculated using 100 random iterations in the presence/absence matrix of OGs. Each blue dot represents one iteration, and the black line is the smooth curve of regression calculated using a generalized additive model (gam) with a cubic spline under the formula y ~ s(x, bs = “cs”). (B) Cumulative (green dots) and non-cumulative (blue dots) numbers of orthologs shared as the number of species increases in the range 2 to 172. The Y axis is represented in logarithmic scale to facilitate visualization. Figure S2. Phylogenetic comparisons of the Spirochaetes phylum (I). (A) Co-phylo plot representing the comparison between the phylogeny obtained under the unrooted homogeneous model of evolution (LG+F+I+R10, left side) and the unrooted heterogeneous model of evolution (LG+C20+R10, right side). Red lines connect the same leaves (species) in both trees. (B) Co-phylo plot representing the comparison between the phylogeny obtained under the rooted homogeneous model of evolution (LG+F+I+R10, left side) and the rooted heterogeneous model of evolution (LG+C20+R10, right side). Red lines connect the same leaves (species) in both trees. Figure S3. Phylogenetic comparisons of the Spirochaetes phylum (II). (A) Co-phylo plot representing the comparison between the phylogeny obtained under the rooted homogeneous model of evolution (LG+F+I+R10, left side) and the unrooted homogeneous model of evolution (LG+F+I+R10, right side). Red lines connect the same leaves (species) in both trees. (B) Co-phylo plot representing the comparison between the phylogeny obtained under the rooted heterogeneous model of evolution (LG+C20+R10, left side) and the unrooted heterogeneous model of evolution (LG+C20+R10, right side). Red lines connect the same leaves (species) in both trees. Figure S4. Phylogenetic comparisons of the Sp [file media-1.zip › supplementary data/Figure_S7.png]

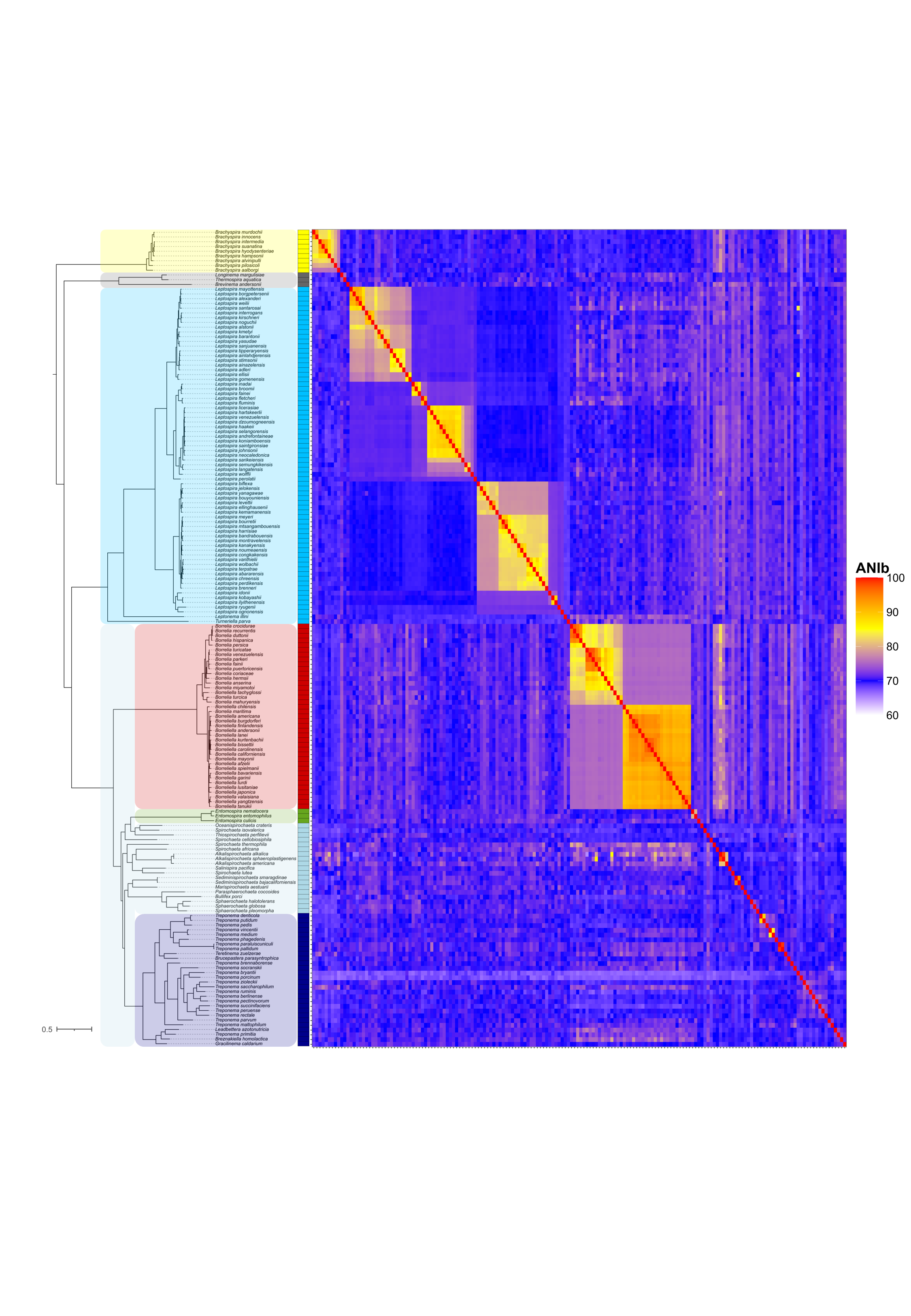

Supplement: Supplement 1 — Figure S1. Pangenome analysis of the phylum Spirochaetes. (A) Pangenome accumulation plot of the Spirochaetes phylum representing the cumulative number of different OGs. This was calculated using 100 random iterations in the presence/absence matrix of OGs. Each blue dot represents one iteration, and the black line is the smooth curve of regression calculated using a generalized additive model (gam) with a cubic spline under the formula y ~ s(x, bs = “cs”). (B) Cumulative (green dots) and non-cumulative (blue dots) numbers of orthologs shared as the number of species increases in the range 2 to 172. The Y axis is represented in logarithmic scale to facilitate visualization. Figure S2. Phylogenetic comparisons of the Spirochaetes phylum (I). (A) Co-phylo plot representing the comparison between the phylogeny obtained under the unrooted homogeneous model of evolution (LG+F+I+R10, left side) and the unrooted heterogeneous model of evolution (LG+C20+R10, right side). Red lines connect the same leaves (species) in both trees. (B) Co-phylo plot representing the comparison between the phylogeny obtained under the rooted homogeneous model of evolution (LG+F+I+R10, left side) and the rooted heterogeneous model of evolution (LG+C20+R10, right side). Red lines connect the same leaves (species) in both trees. Figure S3. Phylogenetic comparisons of the Spirochaetes phylum (II). (A) Co-phylo plot representing the comparison between the phylogeny obtained under the rooted homogeneous model of evolution (LG+F+I+R10, left side) and the unrooted homogeneous model of evolution (LG+F+I+R10, right side). Red lines connect the same leaves (species) in both trees. (B) Co-phylo plot representing the comparison between the phylogeny obtained under the rooted heterogeneous model of evolution (LG+C20+R10, left side) and the unrooted heterogeneous model of evolution (LG+C20+R10, right side). Red lines connect the same leaves (species) in both trees. Figure S4. Phylogenetic comparisons of the Sp [file media-1.zip › supplementary data/Figure_S6.png]

# B

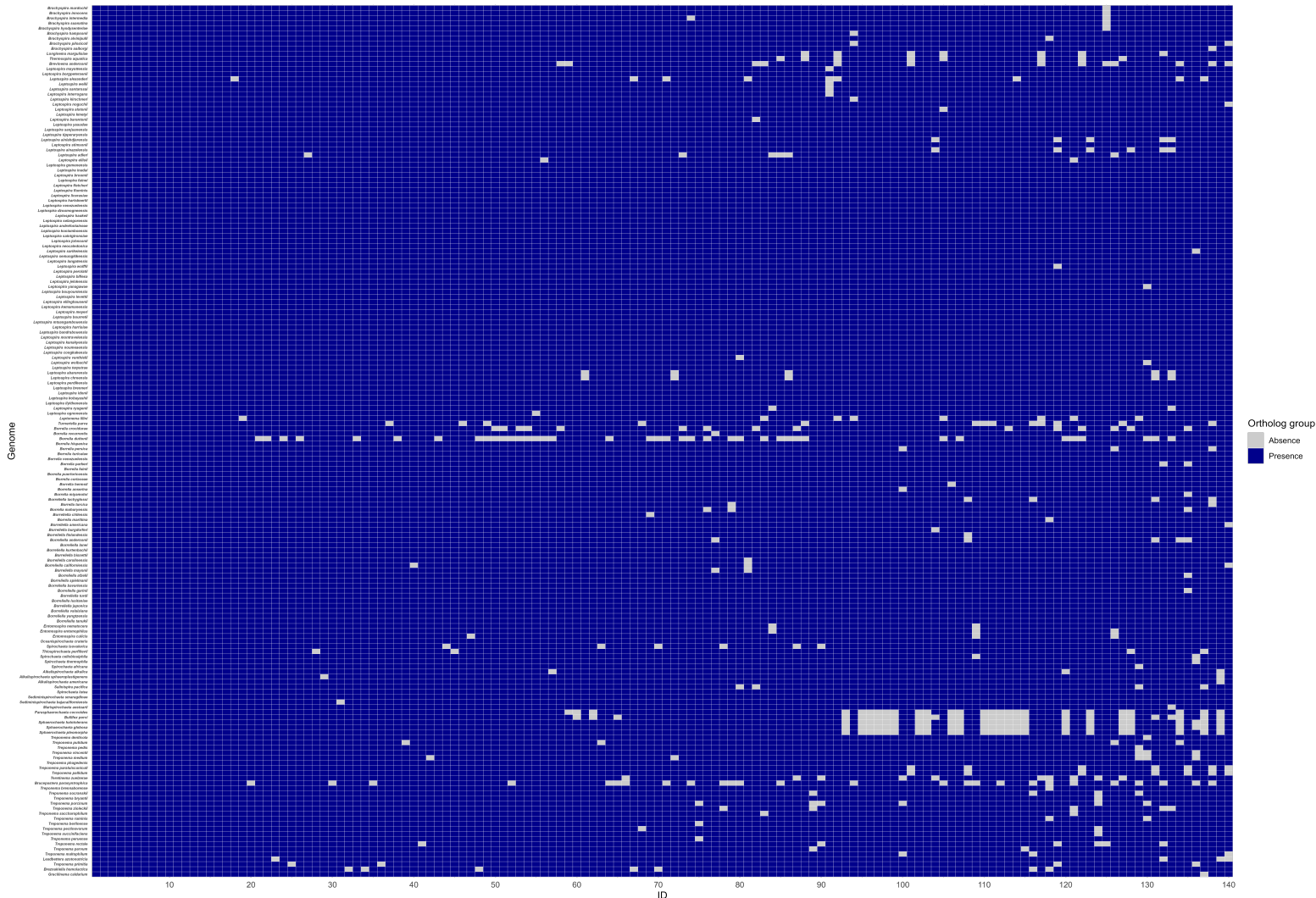

Supplement: Supplement 1 — Figure S1. Pangenome analysis of the phylum Spirochaetes. (A) Pangenome accumulation plot of the Spirochaetes phylum representing the cumulative number of different OGs. This was calculated using 100 random iterations in the presence/absence matrix of OGs. Each blue dot represents one iteration, and the black line is the smooth curve of regression calculated using a generalized additive model (gam) with a cubic spline under the formula y ~ s(x, bs = “cs”). (B) Cumulative (green dots) and non-cumulative (blue dots) numbers of orthologs shared as the number of species increases in the range 2 to 172. The Y axis is represented in logarithmic scale to facilitate visualization. Figure S2. Phylogenetic comparisons of the Spirochaetes phylum (I). (A) Co-phylo plot representing the comparison between the phylogeny obtained under the unrooted homogeneous model of evolution (LG+F+I+R10, left side) and the unrooted heterogeneous model of evolution (LG+C20+R10, right side). Red lines connect the same leaves (species) in both trees. (B) Co-phylo plot representing the comparison between the phylogeny obtained under the rooted homogeneous model of evolution (LG+F+I+R10, left side) and the rooted heterogeneous model of evolution (LG+C20+R10, right side). Red lines connect the same leaves (species) in both trees. Figure S3. Phylogenetic comparisons of the Spirochaetes phylum (II). (A) Co-phylo plot representing the comparison between the phylogeny obtained under the rooted homogeneous model of evolution (LG+F+I+R10, left side) and the unrooted homogeneous model of evolution (LG+F+I+R10, right side). Red lines connect the same leaves (species) in both trees. (B) Co-phylo plot representing the comparison between the phylogeny obtained under the rooted heterogeneous model of evolution (LG+C20+R10, left side) and the unrooted heterogeneous model of evolution (LG+C20+R10, right side). Red lines connect the same leaves (species) in both trees. Figure S4. Phylogenetic comparisons of the Sp [file media-1.zip › supplementary data/Figure_S4.pdf]
